# Prioritizing variants of uncertain significance for reclassification using a rule-based algorithm in inherited retinal dystrophies

**DOI:** 10.1038/s41525-021-00182-z

**Published:** 2021-02-23

**Authors:** Ionut-Florin Iancu, Almudena Avila-Fernandez, Ana Arteche, Maria Jose Trujillo-Tiebas, Rosa Riveiro-Alvarez, Berta Almoguera, Inmaculada Martin-Merida, Marta Del Pozo-Valero, Irene Perea-Romero, Marta Corton, Pablo Minguez, Carmen Ayuso

**Affiliations:** 1 Department of Genetics, Instituto de Investigación Sanitaria–Fundación Jiménez Díaz University Hospital, Universidad Autónoma de Madrid (IIS-FJD, UAM), Madrid, Spain; 2 Center for Biomedical Network Research on Rare Diseases (CIBERER), ISCIII, Madrid, Spain

**Keywords:** Disease genetics, Retinal diseases

## Abstract

Inherited retinal dystrophies (IRD) are a highly heterogeneous group of rare diseases with a molecular diagnostic rate of >50%. Reclassification of variants of uncertain significance (VUS) poses a challenge for IRD diagnosis. We collected 668 IRD cases analyzed by our geneticists using two different clinical exome-sequencing tests. We identified 114 unsolved cases pending reclassification of 125 VUS and studied their genomic, functional, and laboratory-specific features, comparing them to pathogenic and likely pathogenic variants from the same cohort (*N* = 390). While the clinical exome used did not show differences in diagnostic rate, the more IRD-experienced geneticist reported more VUS (*p* = 4.07e-04). Significantly fewer VUS were reported in recessive cases (*p* = 2.14e-04) compared to other inheritance patterns, and of all the genes analyzed, *ABCA4* and *IMPG2* had the lowest and highest VUS frequencies, respectively (*p* = 3.89e-04, *p* = 6.93e-03). Moreover, few frameshift and stop-gain variants were found to be informed VUS (*p* = 6.73e-08 and *p* = 2.93e-06). Last, we applied five pathogenicity predictors and found there is a significant proof of deleteriousness when all score for pathogenicity in missense variants. Altogether, these results provided input for a set of rules that correctly reclassified ~70% of VUS as pathogenic in validation datasets. Disease- and setting-specific features influence VUS reporting. Comparison with pathogenic and likely pathogenic variants can prioritize VUS more likely to be reclassified as causal.

## Introduction

Inherited retinal dystrophies (IRD) are a group of degenerative and progressive diseases caused by primary affection of photoreceptors and retinal pigmentary epithelium cells^1^. Despite their low individual prevalence, together these diseases affect 1 of every 3000–4000 people in the western world^2^. They have high clinical heterogeneity, spanning central (i.e., macular dystrophies), peripheral (i.e., retinitis pigmentosa), and mixed (i.e., cone-rod dystrophies) forms. They include both syndromic disorders (e.g., Usher, Bardet-Biedl, or Joubert) with overlapping phenotypes, as well as non-syndromic forms, all with a wide range of inheritance patterns.

The introduction of next generation-sequencing (NGS) techniques has increased our understanding of IRDs^3,4^. Use of this technology has contributed to the description of an increasing number of causative genes, with 271 identified to date according to RetNet, the Retinal Information Network (https://sph.uth.edu/retnet/). In spite of the rapid pace of discovery, roughly 50% of the studied cases remain unsolved^5,6^. Furthermore, the advent of NGS has also brought with it new challenges, including the classification and interpretation of the thousands of variants detected in a routine analysis. For this task, the American College of Medical Genetics and Genomics (ACMG) and the Association for Molecular Pathology (AMP) offer the most widely used set of guidelines^7^, indicating that variants can be classified as: (i) benign; (ii) likely benign; (iii) of uncertain significance; (iv) likely pathogenic; and (v) pathogenic. Variants of uncertain significance (VUS) account for a large part of genome variability^8,9^ and are the main source of uncertainty in genetic diagnoses. In our experience at the Genetics and Genomics Department of the University Hospital Fundación Jiménez Díaz (HU-FJD), the percentage of unsolved cases pending at least one VUS reclassification is about 17% of all IRD families analyzed. Reclassifying these variants would doubtless have a substantial impact on the clinical management of patients.

There are several approaches that can be used to follow up and reclassify a VUS: (i) in vivo and/or in vitro functional assessment tests to mimic the mutation and observe phenotype changes^10,11^; (ii) cumulative evidence from other cases identified as having the same VUS and phenotype^12^; and (iii) the segregation of the variant within the family. These approaches have obvious limitations, including a delay in diagnosis, which is one of the major challenges in rare diseases, as they postpone appropriate clinical management for an average of five years^13^. Though also limited, other approaches include introducing new annotations such as local population allele frequencies^7^ and periodic reanalysis of genomic tests, which have been shown to significantly increase diagnostic rates^14^.

Numerous collaborative initiatives have been undertaken to boost VUS reclassification in specific diseases. Some of the more noteworthy include InSIGHT, which works to classify gastrointestinal cancer variants; ENIGMA, focused on determining the clinical significance of sequence variants in breast cancer genes; and ClinGEN, a resource dedicated to defining and improving the clinical relevance of genes and variants (https://clinicalgenome.org). Other research groups focus on the adjustment of ACMG/AMP guidelines to gene and disease particularities^15,16^. In addition, several bioinformatics methods have been developed for use in VUS reclassification based on (i) network topology^17^, (ii) data integration^18^, (iii) pathogenicity prediction using conservation and/or structural features^19–27^, and (iv) combined in silico and in vitro analysis^28^.

In this work, we studied a large cohort of IRD patients, providing a comprehensive in-depth report of VUS frequencies according to their molecular, functional, and setting-specific features. The results reveal biases in VUS reporting when diagnosing IRD in our department and allowed us to develop a laboratory algorithm for clinical management of VUS.

## Results

### The frequency of VUS reporting in IRD differs according to the inheritance pattern and causative genes

**Fig. 1:**
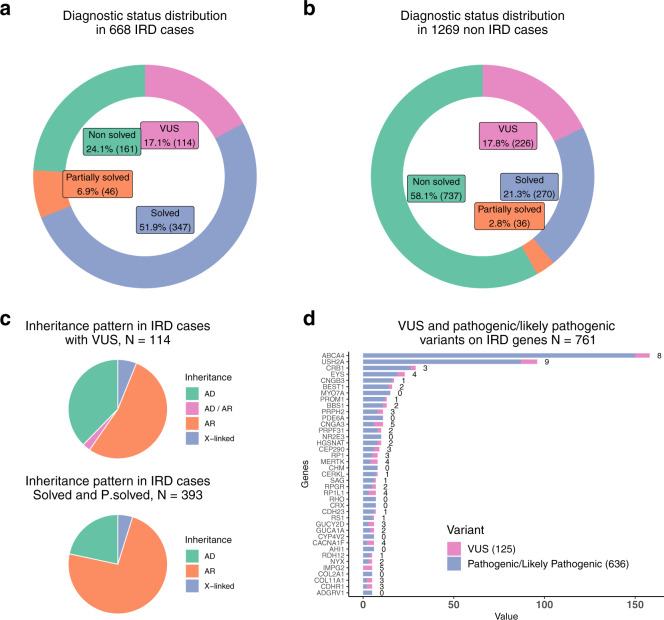
Diagnosis, inheritance, and gene distribution. VUS reporting frequency for IRD (**a**) and non-IRD cases (**b**). For IRD, the inheritance pattern was compared for solved and partially solved cases versus VUS cases (**c**). Four VUS cases have not been included in the plot since an inheritance pattern has not been established for them. (**d**) VUS (number appearing beside VUS diagnoses for each gene) and pathogenic/likely pathogenic variants were compared on IRD genes.

We gathered data on 1937 cases of rare genetic diseases evaluated using targeted panel sequencing in our genetics department over a two-year period; 35% (*n* = 668) were IRD cases (Supplementary Tables 1 and 2). Figure 1a, b show the number of IRD and non-IRD cases as absolute values and percentages of the whole sample, respectively. All cases were classified as solved, partially solved, non-solved cases pending classification of at least one VUS (herein named VUS cases), and non-solved cases. To determine whether the type of disease (the IRD in itself) or our expertize in IRD cases influenced the degree of successful diagnosis of cases, we compared the rates of diagnosis status in the IRD and non-IRD cohorts.

There were significantly more cases of solved and partially solved cases in the IRD (*p*-value = 1.21e-41, *p*-value = 6.91e-05) than non-IRD group, and the IRD group had fewer unsolved cases (*p*-value = 3.17e-47). In contrast, the likelihood of a reported VUS did not differ significantly between IRD and non-IRD.

With regard to the IRD cohort, we first tested whether differences in perceived dominant and recessive inheritance patterns may have influenced VUS reporting. Among IRD cases with dominant inheritance, we found significantly more VUS cases when compared to a reference set of variants formed by pathogenic and likely pathogenic variants reported by the analysts (Fig. 1c, *p*-value = 2.14e-04, and Supplementary Table 3).

We also tested the differential distribution of VUS cases (*N* = 125) and pathogenic and likely pathogenic variants (*N* = 636) in IRD genes (Fig. 1d). In order to determine the mutational load in our IRD cohort, all variants were used. Only two genes were significant outliers within the overall distribution, that is, *IMPG2*, with a higher grade of uncertain diagnosis since all variants identified in this gene were VUS (*p*-value = 6.93e-03), and *ABCA4*, with lower VUS reporting (*p*-value = 3.89e-04, Supplementary Table 4).

### NGS platform type does not impact the uncertainty level while gene panel size and the supervising analyst are associated with differences in VUS reporting frequency

**Fig. 2:**
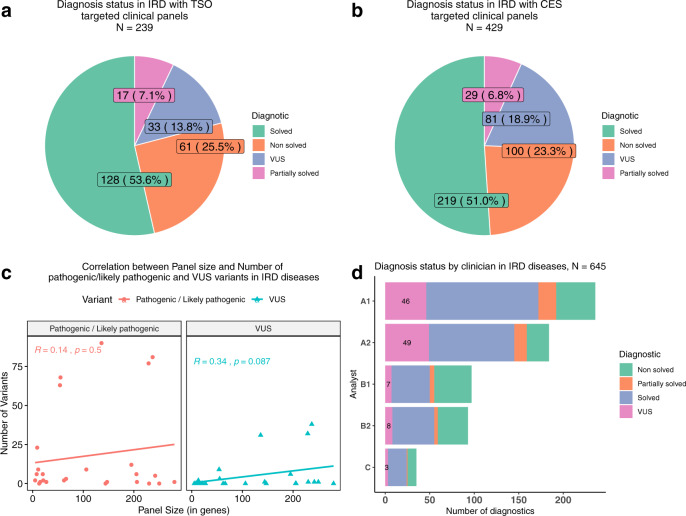
NGS platform, gene panels, and analyst impact. Diagnostic status in IRD using TSO (**a**) and CES clinical exome panels (**b**). Correlation between IRD panels size used and number of VUS/ pathogenic-likely pathogenic variants found (**c**). Diagnosis status by analyst, 23 cases were analyzed by two analysts at the same time and were not considered in this analysis (**d**).

In order to test the impact of different targeted panel-sequencing approaches on VUS reporting, we analyzed the total number of pathogenic and likely pathogenic variants reported and VUS variants reported with each technology. Sequencing coverage metrics do not affect detection accuracy (Supplementary Fig. 1) although they have different variant selection filters, which result on different number of variants to be finally analyzed by the geneticists (Supplementary Fig. 2). Still this does not have an impact on final diagnosis rates as no significant differences in diagnostic rates, including the frequency of VUS cases, were found between the TSO and CES clinical exome systems (Fig. 2a, b and Supplementary Table 5).

We compared the number of VUS reported and pathogenic and likely pathogenic variants reported when applying different virtual panels for IRD cases. Panel’s sizes ranged from 5 to 237 genes. While reference variants showed a small correlation coefficient (*R* = 0.14), a moderate correlation value was found for VUS (*R* = 0.34), and was mainly influenced by the three different virtual panels used in 102 cases with suspicion of any type of IRD (RetNet panel used in 38 VUS cases and a customized proprietary panel used in 33 VUS cases) or more specifically, a non-syndromic IRD panel (31 VUS cases), where 31 VUS of a total of 125 were found (Fig. 2c).

Since VUS reporting is not mandatory in the process of diagnosis, another source of variability in the decision to report VUS is attributable to the geneticist who performs the diagnosis. Here, the degree of analyst expertize and the number of cases studied may influence whether a VUS is reported or not. In our department, of the five geneticists (named in Fig. 2d sorted by their experience in IRD diagnosis), A2 reported significantly more VUS diagnoses (Fig. 2d, *p*-value = 4.07e-04). In contrast, analysts B1 and B2 produced fewer VUS diagnoses (*p*-value = 1.37e-02 and *p*-value = 3.14e-02, respectively). For unsolved cases, we found that analysts A1 and A2 had fewer unsolved diagnoses (*p*-value = 2.67e-02 and *p*-value = 2.22e-04, respectively) and analysts B1 and B2 had more unsolved diagnoses (*p*-value = 2.84e-05 and *p*-value = 7.59e-03, respectively, Supplementary Table 6). All geneticists analyzed a similar proportion of IRD types (Supplementary Table 7).

### VUS reporting frequency depends on gene region but not on gene size nor on essentiality-related gene features

**Fig. 3:**
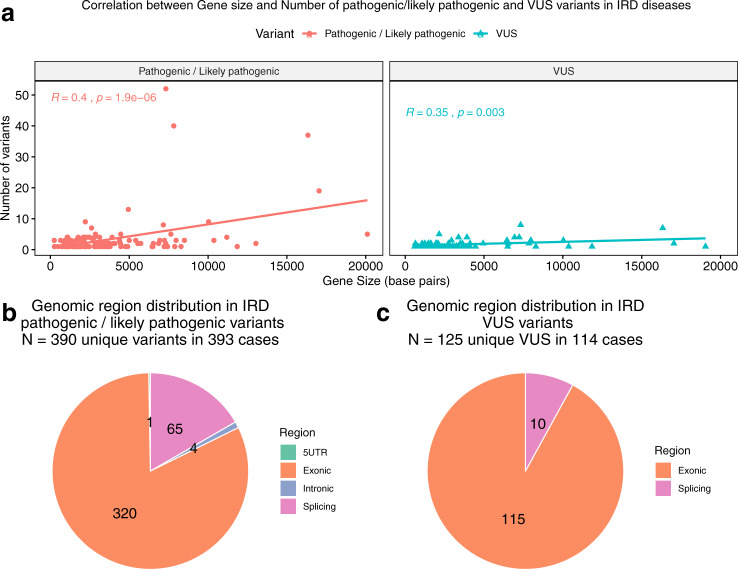
Gene size and genomic region distribution. Correlation between gene size and number of pathogenic/likely pathogenic and VUS variants found (**a**) and genomic region distribution of pathogenic/likely pathogenic variants (**b**) and VUS (**c**).

In order to rule out the influence of gene length on variant reporting, we studied the correlation between gene size and number of VUS reported compared to the reported pathogenic and likely pathogenic variants. The correlation between the exon size (bp of the coding sequences included in the clinical exome panel) and the number of VUS and the reference set of variants was moderate (0.35 for VUS and 0.40 for pathogenic variants), revealing no remarkable differences (Fig. 3a).

Next, we tested whether gene region introduced a bias in the reporting of VUS in unsolved cases. We found that both pathogenic/likely pathogenic variants and VUS were mostly located in exonic regions, specifically 92% of VUS and 82% of the reference set, and VUS showed a significant tendency toward exonic location than pathogenic/likely pathogenic variants (*p*-value = 2.97e-03). No VUS were located in 5’UTR or intronic regions. Splicing regions were more frequent as the site of pathogenic/likely pathogenic variants (16.8%) when compared to VUS (7.9%) (Fig. 3b, c and Supplementary Table 8).

We used ExACpLI score^29^ as an indicator of the likelihood that a gene would be intolerant of a loss of function (LoF) mutation to test whether our selected VUS location would depend on the essentiality of genes. We performed two analyses, first using all variants types in all genes, and second, only LoF variants for all genes. This score did not show any differential distribution for VUS compared to pathogenic/likely pathogenic reported variants for both analyses mentioned before (Supplementary Fig. 3). We also used CCR (constrained coding region) score to map the VUS and pathogenic/likely pathogenic variants reported in our cases^30^. Although no significant differences were found between overall distributions of these scores for VUS and variants with certain degree of pathogenicity, VUS variants appear to have peaks outside the most essential regions, and in causal variants these are less visible (Supplementary Fig. 4).

### Variant pathogenicity predictions and variant consequence may aid in VUS prioritization

**Fig. 4:**
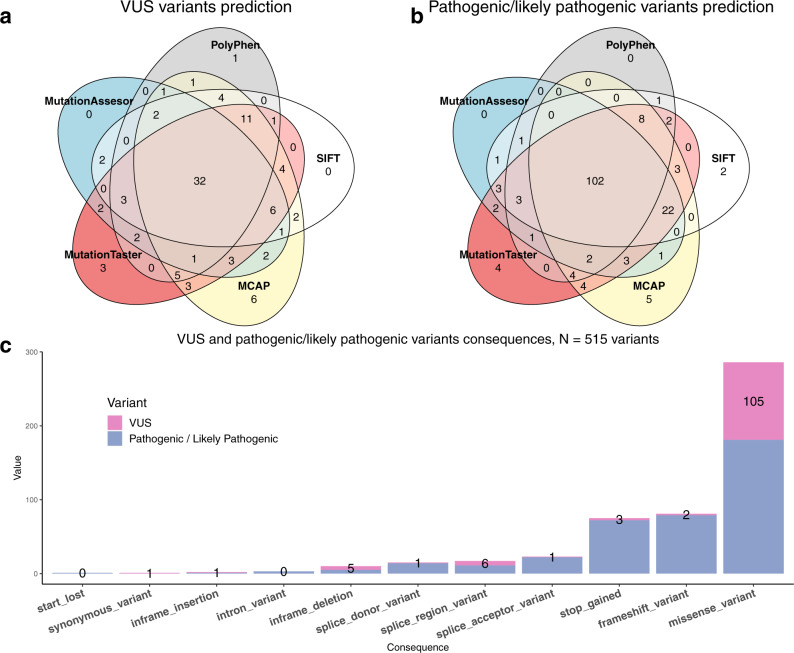
Pathogenicity and variant consequence. Behavior of pathogenicity predictors for VUS variants (**a**) and pathogenic/likely pathogenic (**b**) variants, and variant consequence distribution for pathogenic and VUS (**c**) variants.

Pathogenicity predictions from five popular algorithms (SIFT, PolyPhen, M-CAP, MutationAssessor, and MutationTaster) were calculated to study their ability to predict deleteriousness in pathogenic/likely pathogenic variants and VUS so as to detect any sources of bias. A consensus of 5/5 predictors was obtained for 102/176 missense pathogenic variants (58%), classifying them as deleterious (Fig. 4b). Regarding predictions in VUS, the five predictors overlap in their pathogenicity assessments in 32/105 missense VUS, a significantly lower percentage (30.5%, *p*-value = 1.5e-03) to the one shown for reported causal variants (Fig. 4a and Supplementary Table 9).

**Fig. 5:**
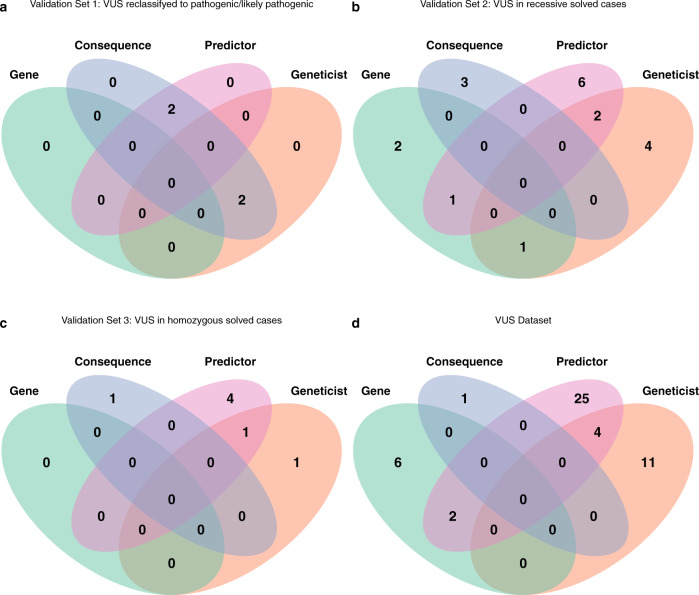
Rule-based algorithm. Applied to VUS reclassified as pathogenic/likely pathogenic dataset (**a**), VUS in recessive solved cases (**b**), VUS in homozygous solved cases (**c**), and applied to VUS dataset (**d**).

Variant consequences were annotated for our set of pathogenic variants and VUS (Fig. 4c) to study their contributions to differential diagnosis. We found that 84% of VUS were missense, which was significantly higher than missense pathogenic/likely pathogenic variants, which represent 46.6% of all reference set of variants. Frameshift and stop-gain variants were significantly less abundant in VUS than in pathogenic/likely pathogenic variants (*p* = 6.73e-08 and *p* = 2.93e-06, Supplementary Table 10). Details on the LoF variants classified as VUS and reported during the diagnosis of the cases can be found in Supplementary Comment 1.

### A rule-based algorithm assists in VUS prioritization

We selected the features that indicates significant differences between reported variants with a certain degree of pathogenicity and VUS reported in unsolved cases, as these may help to detect a bias in VUS reporting. The hypothesis behind this decision is that the VUS more similar to pathogenic/likely pathogenic variants would more likely be causative. Thus, our algorithm is composed of four independent and IRD/cohort-specific rules: (1) VUS is in gene with less VUS diagnostics, *ABCA4*, (2) VUS consequence is frameshift or stop-gain, (3) variant classification was made by the geneticist who reports fewer VUS, and (4) the variant was predicted to be deleterious by the five predictors tested.

For validation, we used three different datasets. First, on completion of the analysis (January 2020), a single geneticist not involved in the initial variant classification reviewed the 125 IRD VUS using the Human Gene Mutation Database (HGMD)^31^ and information present in other databases accessible through VarSome^32^ and ClinVar^9^ in the context of the specific cases. Up to 8 VUS were reclassified, 6 as pathogenic/likely pathogenic and 2 as benign (Supplementary Table 11). We had 117 variants that remained classified as VUS. Our algorithm was thus applied to these six VUS that had been newly reclassified as pathogenic/likely pathogenic. Of these, 4/6 (66%) variants complied with at least one rule, and 2/6 complied with two (Supplementary Table 12). Of the two variants reclassified as benign, only one was selected by a single rule (predictor rule), and none by two or more rules. For this validation, the rules that predicted more variants seemed to be consequence and predictor rules with four and two variants detected, respectively. Of the two variants detected with two rules were detected by the geneticist and consequence rule (Fig. 5a).

In a second validation, we applied our algorithm to a set of VUS found in a second allele for recessive genes together with a likely pathogenic or pathogenic variant in the same gene as first allele (Fig. 5b). In our laboratory practices, these VUS are reclassified as likely pathogenic and were not part of the VUS dataset evaluated. A total of 19 out of 25 (76%) complied with at least one algorithm rule and 4 met the criteria for at least 2 (Supplementary Table 13). In this validation, the rules that selected the most variants were the predictor rule and geneticist-rule with nine and seven variants, respectively. The other two rules performed similarly, with four, and three variants detected by each rule individually (gene-, and consequence rules, respectively). The combinations of rules detecting the four variants with more than one rule were the geneticist and predictor rule with two variants, gene and predictor rule with one, and gene and analyst rule detecting the remaining variant.

For the third validation, we applied our rules to 12 variants classified as VUS by ACMG but reclassified during the initial diagnosis of the cases as pathogenic, as being in homozygosity in 12 cases (Fig. 5c). A total of 7/12 (58.3%) variants were selected by at least one rule and 1/12 (8.3%) selected by two rules. The rule that selected the highest number of variants was the predictor rule, with five variants. The rules that selected the lowest number of variants were the geneticist and consequence rule with two and one variants, respectively. The predictor and geneticist rules selected the variant complying two rules (Supplementary Table 14). All the three validation datasets represent a 70% of success.

Based on these results, we finally applied our algorithm to the 117 VUS that remained unclassified (Fig. 5d). Of these, 49 (41.5%) complied with at least one rule, and 6 (5%) of them at least 2 rules (Supplementary Table 15). In this case, the rule that selected the most variants individually was again the predictor rule, with 31/117 (26.5%) of the variants, and the analyst rule, with 15/117 (12.8%). The rules that selected the lowest number of variants were the gene rule, with 8/117 (6.8%) and the gene rule, with 1/117 (1%). The best rule combinations for selecting variants were the geneticist-predictor rule with four variants followed by the predictor-gene rule, with two variants.

We selected the 49 prioritized VUS that met at least one rule (six of them meeting two), using these as pathogenic candidates for further analysis. These VUS are described in Supplementary Table 15 and 16 together with additional criteria for their prioritization.

### Reassessment of cases with prioritized VUS

The cases with VUS from the final list of 49 variants prioritized have been reviewed and reassessed. Thus, we have compiled new evidences supporting pathogenicity for 13 out of 49 variants (26.5%), see Supplementary Table 16 for details. These 13 variants have been classified in two groups depending on the strength that new evidences showed: (i) five variants for which the new evidences helped on reclassifying them from VUS to likely pathogenic according to ACMG criteria and (ii) eight variants for which new evidences suggest causality, encouraging a very close follow-up of these VUS and cases.

## Discussion

The central importance of VUS for the diagnosis of genetic diseases is clearly shown by their magnitude in public SNVs databases, representing 42% (281,216/676,366) in ClinVar^9^ and up to 76% (78,705,376/103,457,574) in InterVar, the latter a pre-built database, which partially interprets variants according to the ACMG guidelines^33^. The main limitations of our approach to VUS reclassification include the disease- and center-specificity of our findings. In addition, a curated set of benign variants associated with specific disease would permit the possibility to predict a negative association. Despite this, the methodology proposed is suitable, and can be easily adapted to any clinical setting with or without a disease focus. Nonetheless, a disease-specific focus with a sufficient number of cases is preferable as the results in this type of department will likely be more accurate, avoiding disease bias. The same strategy is used in the different initiatives to curate VUS using different approaches^34,35^.

Regarding the overall composition of our cohort (*N* = 1937), about 35% of the cases (668) present IRDs, followed by polymalformative syndromes (<200 cases), with two other disease families above 100 cases (metabolic and encephalopathies-ID-epilepsy). We chose to focus on IRDs due to the expertize and case mix of our laboratory, and thus the larger sub-cohort available for study; still proportion of unsolved cases pending on a VUS reclassification among diseases does not differ. Our diagnostic ratios (solved/unsolved) in IRDs are consistent with previous studies^36,37^, but differ from the non-IRD sub-cohort, illustrating both the general heterogeneity of rare diseases and the impact of expertize on diagnosis.

Our approach consists of analyzing several laboratory-, disease-, and variant-specific features, searching for those that are significantly different among VUS and pathogenic/likely pathogenic variants. By identifying the informative features (with significant differences), we built a rule -based algorithm to select those VUS more closely resemble causative variants, thus making them more likely to solve the pending cases and therefore deserving closer follow-up. Although the selected rules are specific to our laboratory settings and cohort studied, the features evaluated and the methods presented can be extracted and applied in any setting providing a custom resource for a universal problem that will help the whole community providing new insights about VUS. Focusing on each of the rules, the gene rule was selected based on the differential distribution of pathogenic/likely pathogenic and reported VUS variants over IRD causative genes. Thus, we have seen a significantly lower number of VUS reported in diagnosis involving the *ABCA4* gene, and more diagnoses with informed VUS for *IMPG2*. These results are to be expected for *ABCA4*, a well-known and widely studied gene in IRD, with 1490 variants submitted in HGMD^31^ of which 1300 are disease-linked. The Leiden Open Variation Database (LOVD)^38^ contain a total of 8839 public reports of variants, including 1205 unique variants. Our group has studied this gene extensively^39^. In contrast, *IMPG2* is a substantially small gene, with ~4000 base pairs, and little of its genetic information has been described in variant databases (48 variants in HGMD, 26 linked to disease; only 111 public reports in LOVD, 85 of which are unique variants). As a methodological rule, we selected the two geneticists with a significantly lower rate of VUS reports, that is, B1 and B2, as those with a higher accuracy on VUS classification. Also, these two geneticists with intermediate experience in IRD diagnosis, reported significantly higher unsolved diagnoses. This could suggest that in case of doubt as to whether to report a VUS, geneticists tend to avoid reporting. The results for the combination of all predictors were interesting, as this combination detected a significantly higher number of pathogenic/likely pathogenic variants than VUS variants, when applied to our data. Including it as a rule may help in selecting those VUS with features closer to a variant with a certain level of pathogenicity. As a variant feature, the consequence rule was selected for its implication in gene function. Stop-gain and frameshift variant consequences were significantly higher in pathogenic/likely pathogenic variants when compared to VUS. Although these consequences themselves do not define variant pathogenicity, they could help us when prioritizing VUS.

Our algorithm prioritizes VUS that should be followed-up as being more similar to pathogenic in any of the selected informative features (rules). Still other evidences are needed in order to reclassify them as causative, for instance the finding of a second variant within non-coding regions of the gene in the case of recessive cases or a segregation confirmation for dominant and X-Linked cases. With the list of prioritized VUS in hand, we reassessed the cases and found additional evidences supporting the prioritization for 13 of them (26.5%), six in dominant cases, six in recessive and one in a X-linked case. We comment below on the new evidences found for every particular VUS. In dominant cases, we prioritized a VUS in *HK1*, NM_033497: c.1346 C > T in a case with Retinitis Pigmentosa, found to be a *de novo* variant and reported as likely pathogenic by VarSome. We also prioritized two VUS in *BEST1* gene that affect two cases with Macular Dystrophy; VUS NM_004183.3: c.828C > G, reported as pathogenic in VarSome, and, VUS NM_001139443.1: c.671A > G with other described pathogenic variants in the same amino acid. The inheritance pattern in both BEST1 gene cases match with the clinical history of our patients, and fits the phenotype. Both variants were selected by geneticist- and predictor rule. In gene *IMPG2* (also dominant) we prioritized one other VUS (NM_016247.3: c.2872A > G) in a Macular Dystrophy case, also selected by geneticist- and predictor rule, that cosegregates with the disease in the affected father, and is classified as likely pathogenic in VarSome. Next, we prioritized variant NM_000180.3: c.2705T > C in gene *GUCY2D* in a cone-rod dystrophy case with dominant inheritance pattern, compatible with this case phenotype, and reported as likely pathogenic in VarSome. In a last dominant case, we prioritized variant NM_004698.2: c.1481C > T in gene *PRPF3*, classified as pathogenic in VarSome and ClinVar, and with an inheritance pattern, which agrees with the case clinical history.

Regarding recessive cases, we highlight here a case with two VUS variants in gene *ABCA4:* VUS NM_000350.2: c.5383T > G complying two of our rules, and VUS NM_000350.2: c.2980A > G selected by the gene rule, both classified as likely pathogenic in VarSome, and are consistent with case’s phenotype. Some other interesting recessive cases are reported in gene *USH2A*, first one in a compound heterozygous case with two variants prioritized: NM_206933.2: c.12332C > T and NM_206933.2: c.841A > C, last one classified as likely pathogenic in VarSome, and both segregating in both parents and found to be *in trans*. In a second *USH2A* case we prioritized VUS NM_206933.2: c.7067A > G. Here, the amino acid position affected has been reported to be mutated by a known pathogenic variant. Last variant prioritized in a recessive case is NM_024649.4: c.1205T > C in gene *BBS1*, that cosegregates with the disease in two affected sisters in compound heterozygosity with a pathogenic variant. The remaining prioritized VUS is found in a Retinitis Pigmentosa case in gene *RPGR* (variant: NM_001034853.1: c.379A > G) with a X-Linked inheritance pattern, selected by two rules (gene- and predictor rule). This variant is classified as likely pathogenic in VarSome and ClinVar. Additional comments concerning the assessment of these variants are added as Supplementary Table 16.

In summary, we present a strategy to assist VUS reclassification by prioritizing those VUS more likely to be causative. Our prioritization strategy comes from an exhaustive study of laboratory, cohort, and variant features than can be performed elsewhere. Through the resulting rule-based algorithm, we present a final list of 49 VUS prioritized out of a total of 117. Thus, the algorithm has allowed us to compile new evidences in 13 variants from unsolved cases, 5 of them being reclassified to likely pathogenic. The remaining prioritized VUS will be subject of a close follow-up hoping for a prompt conclusive diagnosis.

## Methods

### Cohort selection

We retrospectively selected all index cases with a sequencing test (*N* = 1937) performed in the Genetics and Genomics Department of the HU-FJD from May 2017 to March 2019. DNA samples were collected from the HU-FJD Biobank.

We established two sub-cohorts: an IRD sub-cohort of 668 index cases, and a non-IRD sub-cohort (*N* = 1269, diseases included, detailed in Supplementary Table 17). According to the diagnostic status set by the geneticists, cases from both cohorts were classified as: (i) solved, where one or two pathogenic variants were found in dominant and recessive cases, respectively, including recessive cases with a pathogenic variant and a VUS, and recessive cases with a homozygous VUS (a certain percentage of error in this classification is expected).; (ii) partially solved, where only one pathogenic variant was found in a recessive gene in cases where this mode of inheritance is suspected. Dominant inheritance was suspected when parents were affected and a recessive one when only the index case or information about relatives was not available; (iii) VUS cases, as those having one VUS in dominant or 1–2 VUS in recessive cases; and (iv) unsolved, where no variants have been informed. Analyses to extract differential distribution of VUS were only performed in the IRD sub-cohort.

### Ethics approval

All patients signed an informed consent before participating. The project was reviewed and approved by the Research Ethics Committee of HU-FJD (approval number PIC065-18_FJD) and fulfill the principles of the Declaration of Helsinki and subsequent reviews.

### Molecular geneticist classification

The five molecular geneticists (herein referred to as geneticists) in charge of IRD diagnosis at the Genetics and Genomics Department of the HU-FJD during the period studied were classified by their experience in IRD diagnosis, measured as the number of cases diagnosed. Geneticists A1 and A2 were the two most experienced, B1 and B2 had an intermediate degree of experience, and C had analyzed the lowest number of cases in the period studied. These geneticists classify variants in accordance with the ACMG guidelines. VUS reporting is not mandatory in genetic diagnosis, so each geneticist reported them based on their own criteria.

### Sequencing test performed

Samples were analyzed using targeted NGS with two different commercial-sequencing targeted panels: TruSightOne Sequencing Panel kit (TSO, Illumina, San Diego, CA), applied to 239 IRD cases; and Clinical Exome Solution-Sequencing Panel kit (CES, Sophia Genetics, Boston, MA), applied to 429 IRD cases. For the non-IRD sub-cohort, TSO was applied to 375 non-IRD cases and CES to 894 cases. TSO targets a total of 4811 genes and CES targets 4493, with 3815 of them overlapping between both panels (Supplementary Fig. 5). Variant calling was performed with Illumina and Sophia Genetics pipelines, respectively, for both single-nucleotide variants (SNVs) and small insertions and deletions (indels). Sophia Genetics also includes detection of copy number variations (CNVs) in their analysis and were used for diagnosis. The diagnosis using TSO did not include CNV analysis as it was not available in the Illumina pipeline. For CES, we have a total of 1.5% solved cases with a pathogenic CNV reported. For TSO we have analyzed CNVs retrospectively in unsolved recessive cases with just one VUS reported using a custom in-house pipeline. No CNV was found in IRD genes that passed quality filters. Both software included exonic, intronic, and UTR analysis.

### Virtual panels used

For IRD cases, a total of 12 virtual panels were used to filter the genes associated with the type of IRD suspected (Supplementary Table 18). In the present IRD diagnosis routine, from the 12 virtual panels, just two are used: a 106-gene panel for syndromic IRDs and a 136-gene panel for non-syndromic IRDs, as listed in Supplementary Table 18.

Variant classification was made using the ACMG/AMP criteria^7^ and the European Society of Human Genetics (ESHG) recommendations^40^. The diagnosis analysis and variant classification were performed by five different geneticists from the genetics department of the HU-FJD.

### Bioinformatics reanalysis

In order to have a homogeneous annotation of all sequencing tests, and to facilitate all analysis to perform, all sequencing data was reanalyzed using a proprietary pipeline for SNVs and CNVs. For SNVs, this pipeline is based on GATK 4.1 variant caller, and uses Burrows-Wheeler Aligner, specifically BWA-MEM algorithm. For CNVs detection, our custom pipeline uses the intersection of four different programs (ExomeDepth, ConVaDING, CODEX2 and panelcn.MOPS)^41–44^. The following databases were used for annotating: (i) allele frequency: ExAC, gnomAD, 1000genomes, Kaviar, SpanishFreq (CSVS), and a local frequency database; (ii) pathogenicity prediction: SIFT, PolyPhen, CADD, LRT, M-CAP, MetaLR, MetaSVM, MutationAssesor, MutationTaster, PROVEAN, and FATHMM; (iii) splicing prediction: ada_score, rf_score, and MaxEntScan; (iv) ClinVar; (v) conservation: phastCons20way and phyloP20way; (vi) gene tolerance to loss of function variants: LoFtool, ExACpLI; (vii) constrained coding regions, gnomAD_CCR; and (viii) loss of heterozygosity, annotated with PLINK. The pipeline is available at https://github.com/TBLabFJD/VariantCallingFJD.

### Statistical analysis of VUS and case features

A Fisher exact test was used to test for the following: differences in the distribution of diagnostic rates in IRD compared with non-IRD diseases; numbers of VUS on dominant versus recessive IRDs; and differences in VUS identification between the CES and TSO clinical exome panels.

We used Pearson correlation coefficient to test the correlation between VUS reporting and gene size (in base pairs) and virtual panel size (in number of genes). The relationship between gene intolerance to loss of function variants (ExACpLI score) and VUS was tested by means of the Wilcoxon rank test. The presence of VUS across constrained coding regions (CCR) was tested with Fisher’s exact test.

To determine the differential distribution of VUS compared to pathogenic variants, a 2 × 2 contingency table was analyzed with Fisher exact test. Genomic location of VUS compared to the location of pathogenic variants was tested with Fisher exact test (2 × 2 contingency table). In all analyses, *p*-values adjusted by FDR < 0.05 were considered significant. We used R for all analyses.

### VUS reviewing and benchmarking

After our analysis, the entire collection of variants previously classified as VUS in our IRD cohort (*N* = 125 variants in 114 cases) was reviewed by an independent analyst using up-to-date annotation collected from VarSome. Up to eight variants were reclassified, that is, six to pathogenic/likely pathogenic and two to benign, based on updated information found in databases, which was not available when first reporting the variants.

In order to test the prediction capacity of our algorithm, we performed three different benchmarking analyses using three different sets of variants: (i) the 8 reclassified VUS after our analysis; (ii) 25 VUS in 25 solved recessive IRD cases in which the VUS was present in trans with a pathogenic variant; and (iii) 12 homozygous VUS in 12 solved cases. According to our laboratory policy, all these cases were considered as solved and VUS as reclassified. We applied our algorithm and measured true-positive predictions for all benchmarking analyses.

New evidences for prioritized cases has been uploaded to ClinVar database (ClinVar Submission Name ID: PV_IISFJD2020).

### Reassessment of the cases with prioritized VUS

Cases with prioritized VUS have been reviewed and reassessed considering new compiled information about: clinical history, segregation studies, phenotype information, inheritance pattern, and information in public databases. The new evidences compiled were added to the ACMG algorithm in order to obtain a variant classification.

### Reporting summary

Further information on research design is available in the Nature Research Reporting Summary linked to this article.

## Supplementary information

Supplementary Information

Reporting Summary

## Data Availability

Data are provided in the supplementary material. Variants from prioritized cases were uploaded to ClinVar database (ClinVar Submission Name ID: PV_IISFJD2020, and ClinVar Accession IDs: SCV001450580.1, SCV001450578.1, SCV001450575.1, SCV001450574.1, SCV001450582.1, SCV001450585.1, SCV001450581.1, SCV001450583.1, SCV001450584.1, SCV001450586.1, SCV001450579.1, SCV001450576.1, and SCV001450577.1).

## Code availability

The pipeline used for the bioinformatics reanalysis is available at https://github.com/TBLabFJD/VariantCallingFJD.
