# Supplementary material for: Prioritizing variants of uncertain significance for reclassification using a rule-based algorithm in inherited retinal dystrophies
Source: NPJ Genom Med. 2021 Feb 23;6:18. doi: 10.1038/s41525-021-00182-z (PMC7902814; doi:10.1038/s41525-021-00182-z)

Prioritization of Variants of Uncertain Significance using a rule-based algorithm in Inherited Retinal Dystrophies, *npj Genomic Medicine*, Iancu IF[1,2], MSc; Avila-Fernandez A[1,2], PhD; Arteche A[1], PhD; Trujillo-Tiebas MJ[1,2], PhD; Riveiro-Alvarez R[1,2], PhD; Almoguera B[1,2], PhD; Martin-Merida I[1,2], PhD; Del Pozo-Valero M[1,2], PhD; Perea-Romero I[1,2], MSc; Corton M[1,2], PhD; Minguez P[1,2] #, PhD; Ayuso C[1,2] #, MD, PhD

1. Department of Genetics, Instituto de Investigación Sanitaria–Fundación Jiménez Díaz University Hospital, Universidad Autónoma de Madrid (IIS-FJD, UAM), Madrid, Spain.

2. Center for Biomedical Network Research on Rare Diseases (CIBERER), ISCIII, Madrid, Spain.

email: cayuso@fjd.es

Supplementary Material

Supplementary Comment 1

Usually Lost of Function variants is a strong evidence for variant classification, still not determining by itself in ACMG rules. This is the case for instance of two of the VUS in our dataset:

1) NM_144499.2: c.359C>A (NP_653082.1: p. Ser120Ter) in the gene *GNAT1*, which is a LoF variant nonsense classified as variant of uncertain clinical significance (VUS) with high frequency in carriers of latino population (https://gnomad.broadinstitute.org/variant/3-50231006-C-A?dataset=gnomad_r2_1) and with unknown inheritance pattern according to our family records. Besides, the same variant has been reported in ClinVar as VUS. As the evidence reported (in ClinVar) explains: *"The current clinical and genetic evidence is not sufficient to establish whether* loss-of-function variants in *GNAT1 cause* the disease.*"* (https://www.ncbi.nlm.nih.gov/clinvar/variation/855140/).

2) NM_031935.2: c.14609-1G>A, in gene *HMNC1* which is not described in databases, and has no population frequencies. Besides, no inheritance pattern is available in our records. For this variant, alteration of splicing is predicted by *in-silico* programs (*Alamut 2.0 Splicing Prediction Module*), however, no causal splicing variants in this gene are described in HGMD. Therefore, it is considered a VUS following ACMG Classification (PM2 - Moderate and PP3 - Supporting).

Supplementary Table 1. Sex distribution in our IRD cohort,
and by IRD type (Retinitis Pigmentosa - RP and Macular Dystrophy - MD)

			**Sex**	
			Male	Female
		Total	356 (53,3%)	312 (46,7%)
IRD type	RP	427 (63,9%)	235 (55%)	192 (45%)
	MD	241 (36,1%)	121 (50,2%)	120 (49,8%)

Supplementary Table 2. Demographic distribution of our IRD cohort, and by IRD type (Retinitis Pigmentosa - RP and Macular Dystrophy - MD)

	IRD Type		
**Autonomous community**	**Macular Dystrophy**	**Retinitis Pigmentosa**	**Total**
Andalusia	6	13	19
Aragon	2	2	4
Principality of Asturias	0	2	2
Canary Islands	11	8	19
Cantabria	7	15	22
Castilla-La Mancha	10	21	31
Castille and Leon	1	9	10
Catalonia	0	2	2
Community of Madrid	128	208	336
Valencian Community	0	3	3
Extremadura	1	4	5
Galicia	0	1	1
Balearic Islands	6	14	20
Chartered Community of Navarre	0	1	1
Basque Country	32	43	75
Region of Murcia	17	30	47
Unknown	20	51	71

Supplementary Table 3. Numbers and adjusted p-values (FDR) used for inheritance comparisons. Two one-sided fisher test were performed (greater and less).

Inheritance	VUS	Solved and Partially Solved	FDR greater	FDR less
**AD**	43	85	2,13E-03	1,00E+00
**AD / AR**	3	0	2,23E-02	1,00E+00
**AR**	62	288	1,00E+00	2,14E-04
**X-linked**	7	19	4,84E-01	1,00E+00

Supplementary Table 4. Numbers and adjusted p-values (FDR) used for gene comparisons.

Gene	VUS	Partially solved	Solved	FDR
**ABCA4**	8	12	138	3,89E-04
**ABCC6**	0	0	1	1,00E+00
**ACO2**	0	0	3	1,00E+00
**ADGRV1**	0	1	4	1,00E+00
**ADIPOR1**	1	0	0	5,50E-01
**AHI1**	0	0	6	1,00E+00
**AIPL1**	2	0	0	3,79E-01
**ATF6**	0	0	2	1,00E+00
**BBS1**	2	1	10	1,00E+00
**BBS12**	0	0	2	1,00E+00
**BBS9**	0	1	2	1,00E+00
**BEST1**	2	0	14	1,00E+00
**C1QTNF5**	0	0	2	1,00E+00
**C2orf71**	1	1	0	8,13E-01
**C8orf37**	0	0	2	1,00E+00
**CA4**	1	0	0	5,50E-01
**CABP4**	0	1	0	1,00E+00
**CACNA1F**	4	0	2	2,47E-01
**CDH23**	1	0	6	1,00E+00
**CDHR1**	3	0	2	3,79E-01
**CEP290**	3	2	4	5,60E-01
**CERKL**	1	1	6	1,00E+00
**CHM**	0	0	8	9,25E-01
**CLN3**	0	0	2	1,00E+00
**CLN8**	0	0	2	1,00E+00
**CNGA1**	0	0	2	1,00E+00
**CNGA3**	5	0	6	3,79E-01
**CNGB1**	2	1	1	5,50E-01
**CNGB3**	1	2	14	8,61E-01
**COL11A1**	3	0	2	3,79E-01
**COL2A1**	0	0	5	1,00E+00
**COL9A1**	1	0	0	5,50E-01
**CRB1**	3	4	22	1,00E+00
**CRX**	0	0	7	1,00E+00
**CYP4V2**	0	0	6	1,00E+00
**DHDDS**	0	0	2	1,00E+00
**EYS**	4	2	17	1,00E+00
**FAM161A**	0	0	2	1,00E+00
**FRMD7**	0	0	1	1,00E+00
**FSCN2**	3	0	0	1,80E-01
**FZD4**	0	0	2	1,00E+00
**GDF6**	1	0	0	5,50E-01
**GNAT1**	1	0	0	5,50E-01
**GNAT2**	0	0	2	1,00E+00
**GPR179**	0	0	2	1,00E+00
**GRM6**	0	0	2	1,00E+00
**GUCA1A**	2	0	4	7,78E-01

**GUCA1B**	1	0	0	5,50E-01
**GUCY2D**	3	1	2	5,50E-01
**HGSNAT**	2	0	8	1,00E+00
**HK1**	1	0	1	8,13E-01
**HMCN1**	1	0	0	5,50E-01
**IFT172**	0	0	2	1,00E+00
**IMPDH1**	2	0	2	5,50E-01
**IMPG2**	5	0	0	6,93E-03
**KCNV2**	1	0	1	8,13E-01
**KLHL7**	1	0	0	5,50E-01
**MAK**	0	0	2	1,00E+00
**MERTK**	4	0	4	3,79E-01
**MFRP**	1	0	0	5,50E-01
**MKS1**	1	0	0	5,50E-01
**MT-ND4**	0	0	1	1,00E+00
**MYO7A**	0	3	12	5,50E-01
**NMNAT1**	0	0	3	1,00E+00
**NPHP4**	0	0	2	1,00E+00
**NR2E3**	0	0	10	9,46E-01
**NRL**	0	0	2	1,00E+00
**NYX**	2	0	3	6,08E-01
**OAT**	0	0	2	1,00E+00
**OFD1**	1	0	2	9,75E-01
**OPA1**	1	0	1	8,13E-01
**OPN1LW**	0	0	1	1,00E+00
**OPN1SW**	0	0	1	1,00E+00
**OTC**	0	0	1	1,00E+00
**PANK2**	1	0	0	5,50E-01
**PAX6**	1	0	0	5,50E-01
**PCDH15**	1	1	2	1,00E+00
**PDE6A**	0	1	10	7,04E-01
**PDE6B**	1	1	0	8,13E-01
**PDE6G**	0	0	2	1,00E+00
**PDZD7**	1	0	0	5,50E-01
**PEX1**	0	0	2	1,00E+00
**PEX6**	0	0	2	1,00E+00
**PNPLA6**	1	0	0	5,50E-01
**PRCD**	0	0	2	1,00E+00
**PROM1**	1	1	11	1,00E+00
**PRPF3**	1	0	0	5,50E-01
**PRPF31**	2	0	8	1,00E+00
**PRPF6**	1	0	0	5,50E-01
**PRPF8**	2	0	1	5,50E-01
**PRPH2**	3	0	8	9,75E-01
**PRPS1**	0	0	1	1,00E+00
**RAX2**	1	0	0	5,50E-01
**RBP3**	0	0	2	1,00E+00
**RDH12**	1	0	4	1,00E+00
**RDH5**	0	0	2	1,00E+00
**RGR**	0	0	3	1,00E+00

**RGS9**	0	1	0	1,00E+00
**RHO**	0	0	7	1,00E+00
**RLBP1**	0	0	4	1,00E+00
**RP1**	3	0	5	5,50E-01
**RP1L1**	4	1	2	3,79E-01
**RP2**	0	0	4	1,00E+00
**RPE65**	0	1	2	1,00E+00
**RPGR**	2	0	5	8,53E-01
**RPGRIP1L**	0	1	0	1,00E+00
**RS1**	1	0	5	1,00E+00
**SAG**	1	0	6	1,00E+00
**SLC24A1**	0	0	4	1,00E+00
**SNRNP200**	0	0	4	1,00E+00
**SPATA7**	1	0	2	9,75E-01
**TCTN1**	1	0	0	5,50E-01
**TOPORS**	0	0	1	1,00E+00
**TRPM1**	0	0	4	1,00E+00
**TSPAN12**	0	0	2	1,00E+00
**TTPA**	0	0	2	1,00E+00
**TULP1**	0	0	2	1,00E+00
**UNC119**	1	0	0	5,50E-01
**USH1C**	0	0	2	1,00E+00
**USH1G**	0	0	2	1,00E+00
**USH2A**	9	4	83	5,50E-01
**VPS13B**	0	0	2	1,00E+00
**WDR19**	0	1	0	1,00E+00
**WFS1**	0	0	2	3,79E-01

Supplementary Table 5. Numbers and adjusted p-values (FDR) used for panel type comparisons.

	Panel type		
	**TSO**	**CES**	**FDR**
**VUS**	33	82	4,31E-01
**Partially solved**	17	28	8,74E-01
**Solved**	128	219	7,63E-01
**Non solved**	61	100	7,63E-01

Supplementary Table 6. Numbers and adjusted p-values (FDR) used for geneticist comparisons.
Two one-sided fisher test were performed (greater and less) for each diagnostic (VUS, Solved, Partially Solved and Non solved).

					LESS			
**Analyst**	**VUS**	**Solved**	**Partially Solved**	**Non Solved**	**FDR_vus**	**FDR_solved**	**FDR_pSolved**	**FDR_nonSolved**
**A2**	49	96	14	25	1,00E+00	8,76E-01	9,11E-01	2,22E-04
**A1**	46	126	20	44	1,00E+00	8,76E-01	9,11E-01	2,67E-02
**C**	3	21	1	10	1,84E-01	8,76E-01	6,34E-01	1,00E+00
**B1**	7	43	5	42	1,37E-02	3,90E-01	6,34E-01	1,00E+00
**B2**	8	47	4	34	3,14E-02	8,76E-01	6,34E-01	1,00E+00

Greater			
FDR_vus	FDR_solved	FDR_pSolved	FDR_nonSolved
4,07E-04	7,59E-01	9,23E-01	1,00E+00
3,92E-01	6,38E-01	8,98E-01	1,00E+00
9,99E-01	6,38E-01	9,23E-01	6,48E-01
9,99E-01	9,59E-01	9,23E-01	2,84E-05
9,99E-01	7,89E-01	9,23E-01	7,59E-03

Supplementary Table 7. Proportion of subpanels analyzed by each geneticist by IRD type (Retinitis Pigmentosa - RP and Macular Dystrohpy - MD). Adjusted p-values are included (FDR).

Geneticist	Total Cases	RP cases	MD cases	p-values	FDR
A1	236	155	81	0.5011414	0.6013697
A2	184	122	62	0.4709176	0.6013697
B1	97	66	31	0.423594	0.6013697
B2	93	48	45	0.01019038	0.0611423
C	35	20	15	0.4697761	0.6013697
Unspecifyed	23	16	7	0.6624136	0.6624136

Supplementary Table 8. Numbers and adjusted p-values (FDR) used for gene region comparisons.

Gene Region	VUS	Pathogenic	FDR
**5UTR**	0	1	1,00E+00
**EXONIC**	115	320	2,97E-03
**INTRONIC**	0	4	7,70E-01
**SPLICING**	10	65	5,40E-02

Supplementary Table 9. Numbers and adjusted p-values (FDR) used for pathogenicity predictors comparisons.

Predictors combination	VUS	Pathogenic / likely pathogenic	FDR
**SIFT**	0	2	9,50E-01
**PolyPhen**	1	0	6,98E-01
**MutationAssessor_pred**	0	0	1,00E+00
**MutationTaster_pred**	3	4	9,50E-01
**M.CAP_pred**	6	5	6,98E-01
**SIFT&PolyPhen**	0	1	1,00E+00
**SIFT&MutationAssessor_pred**	2	1	6,98E-01
**SIFT&MutationTaster_pred**	0	0	1,00E+00
**SIFT&M.CAP_pred**	2	0	6,66E-01
**PolyPhen&MutationAssessor_pred**	0	0	1,00E+00
**PolyPhen&MutationTaster_pred**	0	0	1,00E+00
**PolyPhen&M.CAP_pred**	1	0	6,98E-01
**MutationAssessor_pred&MutationTaster_pred**	2	2	9,50E-01
**MutationAssessor_pred&M.CAP_pred**	2	1	6,98E-01
**MutationTaster_pred&M.CAP_pred**	3	4	9,50E-01
**SIFT&PolyPhen&MutationAssessor_pred**	0	1	1,00E+00
**SIFT&PolyPhen&MutationTaster_pred**	1	2	1,00E+00
**SIFT&PolyPhen&M.CAP_pred**	4	0	2,51E-01
**SIFT&MutationAssessor_pred&MutationTaster_pred**	0	3	9,50E-01
**SIFT&MutationAssessor_pred&M.CAP_pred**	1	0	6,98E-01
**SIFT&MutationTaster_pred&M.CAP_pred**	4	3	6,98E-01
**PolyPhen&MutationAssessor_pred&MutationTaster_pred**	2	1	6,98E-01
**PolyPhen&MutationAssessor_pred&M.CAP_pred**	1	0	6,98E-01
**PolyPhen&MutationTaster_pred&M.CAP_pred**	5	4	6,98E-01
**MutationAssessor_pred&MutationTaster_pred&M.CAP_pred**	3	3	9,50E-01
**SIFT&PolyPhen&MutationAssessor_pred&MutationTaster_pred**	3	3	9,50E-01
**SIFT&PolyPhen&MutationAssessor_pred&M.CAP_pred**	2	0	6,66E-01
**SIFT&PolyPhen&MutationTaster_pred&M.CAP_pred**	11	8	5,01E-01
**SIFT&MutationAssessor_pred&MutationTaster_pred&M.CAP_pred**	6	22	6,66E-01
**PolyPhen&MutationAssessor_pred&MutationTaster_pred&M.CAP_pred**	1	2	1,00E+00
**SIFT&PolyPhen&MutationAssessor_pred&MutationTaster_pred&M.CAP_pred**	32	102	1,51E-03

Supplementary Table 10. Numbers and adjusted p-values (FDR) used for variant consequences comparisons.

	Variant		
**Variant consequence**	**VUS**	**Pathogenic / likely pathogenic**	**FDR**
**frameshift_variant**	2	79	6,73E-08
**inframe_deletion**	5	5	1,51E-01
**inframe_insertion**	1	1	5,23E-01
**intron_variant**	0	3	1,00E+00
**missense_variant**	105	182	3,20E-13
**splice_acceptor_variant**	1	22	6,25E-02
**splice_donor_variant**	1	14	2,43E-01
**splice_region_variant**	6	11	3,64E-01
**start_lost**	0	1	1,00E+00
**stop_gained**	3	72	2,93E-06
**synonymous_variant**	1	0	3,64E-01

Supplementary Table 11. Variants reclassifyed after reviewing all 125 VUS Dataset. 8 variants were reclassifyed; 6 to pathogenic/likely pathogenic and 2 to benign. Initial evaluation and reviewing date are included, together with links to papers when available.

Gene	HGVSc	HGVSp	Variant classification	ACMG Criteria
CACNA1F	NM_005183.2:c.4118T>C	NP_005174.2:p.Val1373Ala	Benign	PP1 Supporting; PP3 Supporting; BS1 Strong; BS2 Strong;
MERTK	NM_006343.2:c.756A>G	NP_006334.2:p.Pro252=	Benign	BA1 Stand Alone; BP4 Supporting; BP6 Moderate
BBS1	NM_024649.4:c.1645G>T	NP_078925.3:p.Glu549Ter	Pathogenic	PVS1 Very Strong; PM2 Moderate; PP3 Supporting; PP5 Strong
CNGA3	NM_001298.2:c.1679C>T	NP_001289.1:p.Ser560Leu	Likely Pathogenic	PM1 Moderate; PM2 Moderate; PP2 Supporting; PP3 Supporting
EYS	NM_001142800.1:c.9368dup	NP_001136272.1:p.Asn3123LysfsTer3	Pathogenic	PVS1 Very Strong; PM2 Moderate; PP3 Supporting
MFRP	NM_031433.3:c.955C>T	NP_113621.1:p.Gln319Ter	Pathogenic	PVS1 Very Strong; PM2 Moderate; PP3 Supporting; PP5 Supporting
PCDH15	NM_001142763.1:c.4936_4939dup	NP_001136235.1:p.Arg1647AsnfsTer5	Pathogenic	PVS1 Very Strong; PM2 Moderate; PP3 Supporting
TCTN1	NM_001082538.2:c.472+1G>A	-	Pathogenic	PVS1 Very Strong; PM2 Moderate; PP3 Supporting

References	First evaluated	Review date
-	July 2017	January 2020
-	January 2018	January 2020
28492532; 22410627; 21517826; 24746959;16327777; 21642631; 12118255; 15770229	February 2018	January 2020
-	February 2019	January 2020
-	October 2017	January 2020
-	May 2018	January 2020
-	October 2017	January 2020
-	March 2018	January 2020

Supplementary Table 12. First validation set. VUS reclassified to pathogenic/likely pathogenic and benign variants.

			Rules					
**Gene**	**HGVSc**	**HGVSp**	**Gene**	**Geneticist**	**Consequence**	**Predictor**	**Variant**	**Rules**
CACNA1F	NM_005183.2:c.4118T>C	NP_005174.2:p.Val1373Ala	0	0	0	1	Benign	1
MERTK	NM_006343.2:c.756A>G	NP_006334.2:p.Pro252=	0	0	0	0	Benign	0
BBS1	NM_024649.4:c.1645G>T	NP_078925.3:p.Glu549Ter	0	0	1	0	Pathogenic	1
CNGA3	NM_001298.2:c.1679C>T	NP_001289.1:p.Ser560Leu	0	0	0	0	Likely pathogenic	0
EYS	NM_001142800.1:c.9368dup	NP_001136272.1:p.Asn3123LysfsTer3	0	1	1	0	Pathogenic	2
MFRP	NM_031433.3:c.955C>T	NP_113621.1:p.Gln319Ter	0	0	1	0	Pathogenic	1
PCDH15	NM_001142763.1:c.4936_4939dup	NP_001136235.1:p.Arg1647AsnfsTer5	0	1	1	0	Pathogenic	2
TCTN1	NM_001082538.2:c.472+1G>A	-	0	0	0	0	Pathogenic	0

Supplementary Table 13. Second validation set. VUS in recessive solved cases with a likely pathogenic or pathogenic variant.

			Rules					
**Gene**	**HGVSc**	**HGVSp**	**Gene**	**Geneticist**	**Consequence**	**Predictor**	**Variant**	**Rules**
ABCA4	NM_000350.2:c.2878G>A	NP_000341.2:p.Ala960Thr	1	1	0	0	Recessive_VUS	2
ABCA4	NM_000350.2:c.5981G>A	NP_000341.2:p.Gly1994Glu	1	0	0	1	Recessive_VUS	2
ABCA4	NM_000350.3:c.4539+2064C>T	-	1	0	0	0	Recessive_VUS	1
ABCA4	NM_000350.3:c.5603A>T	NP_000341.2:p.Asn1868Ile	1	0	0	0	Recessive_VUS	1
ADGRV1	NM_032119.3:c.15751T>A	NP_115495.3:p.Phe5251Ile	0	0	0	0	Recessive_VUS	0
CLN8	NM_018941.3:c.200C>T	NP_061764.2:p.Ala67Val	0	0	0	1	Recessive_VUS	1
CNGA3	NM_001298.2:c.1343A>G	NP_001289.1:p.Lys448Arg	0	0	0	0	Recessive_VUS	0
CNGB3	NM_019098.4:c.1208G>A	NP_061971.3:p.Arg403Gln	0	1	0	0	Recessive_VUS	1
CRB1	NM_201253.2:c.3946_3949del	NP_957705.1:p.Leu1316ThrfsTer24	0	0	1	0	Recessive_VUS	1
EYS	NM_001292009.1:c.159C>G	NP_001278938.1:p.Phe53Leu	0	1	0	0	Recessive_VUS	1
EYS	NM_001142800.1:c.1461A>T	NP_001136272.1:p.Gly487=	0	0	0	0	Recessive_VUS	0
GUCY2D	NM_000180.3:c.2591C>T	NP_000171.1:p.Ala864Val	0	1	0	0	Recessive_VUS	1
HGSNAT	NM_152419.2:c.1610del	NP_689632.2:p.Leu537ProfsTer27	0	0	1	0	Recessive_VUS	1
HGSNAT	NM_152419.2:c.372-2A>G	-	0	0	0	0	Recessive_VUS	0
HGSNAT	NM_152419.2:c.1843G>A	NP_689632.2:p.Ala615Thr	0	0	0	0	Recessive_VUS	0
MYO7A	NM_000260.3:c.2542C>T	NP_000251.3:p.Arg848Trp	0	0	0	1	Recessive_VUS	1
PEX1	NM_000466.2:c.1729C>T	NP_000457.1:p.Arg577Cys	0	0	0	0	Recessive_VUS	0
PEX6	NM_000287.3:c.2626C>T	NP_000278.3:p.Arg876Trp	0	1	0	1	Recessive_VUS	2
PROM1	NM_006017.2:c.1406C>T	NP_006008.1:p.Pro469Leu	0	0	0	1	Recessive_VUS	1
USH2A	NM_206933.2:c.14147A>T	NP_996816.2:p.Asn4716Ile	0	1	0	1	Recessive_VUS	2
USH2A	NM_206933.2:c.5858C>G	NP_996816.2:p.Ala1953Gly	0	1	0	0	Recessive_VUS	1
USH2A	NM_206933.2:c.8693A>C	NP_996816.2:p.Tyr2898Ser	0	0	0	1	Recessive_VUS	1
USH2A	NM_206933.2:c.1531G>A	NP_996816.2:p.Glu511Lys	0	0	0	1	Recessive_VUS	1
USH2A	NM_206933.2:c.1938G>C	NP_996816.2:p.Gly647Arg	0	0	0	1	Recessive_VUS	1
VPS13B	NM_017890.4:c.11598del	NP_060360.3:p.Glu3867LysfsTer11	0	0	1	0	Recessive_VUS	1

Supplementary Table 14. Third validation set. VUS in homozygous solved cases.

			Rules					
**Gene**	**HGVSc**	**HGVSp**	**Gene**	**Geneticist**	**Consequence**	**Predictor**	**Variant**	**Rules**
USH2A	NM_206933.2:c.7415A>C	NP_996816.2:p.Gln2472Pro	0	1	0	1	Homozygous_VUS	2
MYO7A	NM_000260.3:c.1846C>T	NP_000251.3:p.Arg616Trp	0	0	0	1	Homozygous_VUS	1
RLBP1	NM_000326.4:c.525G>C	NP_000317.1:p.Glu175Asp	0	0	0	0	Homozygous_VUS	0
SAG	NM_000541.4:c.769G>A	NP_000532.2:p.Asp257Asn	0	0	0	0	Homozygous_VUS	0
RGR	NM_001012720.1: c.824_825insCAATGAG	NP_001012738.1:p.Ile276AsnfsTer80	0	0	1	0	Homozygous_VUS	1
NMNAT1	NM_001297778.1:c.769G>A	NP_001284707.1:p.Glu257Lys	0	0	0	0	Homozygous_VUS	0
RBP3	NM_002900.2: c.3618_3638del	NP_002891.1: p.Trp1206_Pro1213delinsCys	0	1	0	0	Homozygous_VUS	1
GNAT2	NM_005272.3:c.107T>G	NP_005263.1:p.Leu36Arg	0	0	0	1	Homozygous_VUS	1
BBS12	NM_152618.2:c.1627G>A	NP_689831.2:p.Glu543Lys	0	0	0	1	Homozygous_VUS	1
CRB1	NM_201253.2:c.1910C>T	NP_957705.1:p.Pro637Leu	0	0	0	1	Homozygous_VUS	1
GPR179	NM_001004334.2:c.6802C>T	NP_001004334.2:p.Pro2268Ser	0	0	0	0	Homozygous_VUS	0
ATF6	NM_007348.3:c.909+5G>A	-	0	0	0	0	Homozygous_VUS	0

Supplementary Table 15. VUS dataset. Total list with 117 VUS tested, and the 49 VUS prioritized by our rule-based algorithm (selected by at least one rule).

Gene	HGVSc	HGVSp	Gene-rule	Geneticist-rule	Consequence-rule	Predictor-rule	Variant	Rules
ABCA4	NM_000350.2:c.3783T>G	NP_000341.2:p.Ser1261Arg	1	0	0	1	VUS	2
ABCA4	NM_000350.2:c.5383T>G	NP_000341.2:p.Leu1795Val	1	0	0	1	VUS	2
RPGR	NM_001034853.1:c.379A>G	NP_001030025.1:p.Arg127Gly	0	1	0	1	VUS	2
BEST1	NM_001139443.1:c.671A>G	NP_001132915.1:p.Tyr224Cys	0	1	0	1	VUS	2
BEST1	NM_004183.3:c.828C>G	NP_004174.1:p.Phe276Leu	0	1	0	1	VUS	2
IMPG2	NM_016247.3:c.2872A>G	NP_057331.2:p.Ser958Gly	0	1	0	1	VUS	2
GNAT1	NM_144499.2:c.359C>A	NP_653082.1:p.Ser120Ter	0	0	1	0	VUS	1
MKS1	ENST00000393119.2:c.1424G>A	ENSP00000376827.2:p.Arg475His	0	0	0	1	VUS	1
HK1	NM_033497.2:c.1346C>T	NP_277032.1:p.Ser449Leu	0	0	0	1	VUS	1
GUCY2D	NM_000180.3:c.2705T>C	NP_000171.1:p.Val902Ala	0	0	0	1	VUS	1
PAX6	NM_000280.4:c.1189A>T	NP_000271.1:p.Ile397Phe	0	0	0	1	VUS	1
PRPH2	NM_000322.4:c.112G>A	NP_000313.2:p.Gly38Arg	0	0	0	1	VUS	1
ABCA4	NM_000350.2:c.1319A>G	NP_000341.2:p.Tyr440Cys	1	0	0	0	VUS	1
ABCA4	NM_000350.2:c.2054C>T	NP_000341.2:p.Thr685Ile	1	0	0	0	VUS	1
ABCA4	NM_000350.2:c.2353C>T	NP_000341.2:p.Arg785Cys	1	0	0	0	VUS	1
ABCA4	NM_000350.2:c.2980A>G	NP_000341.2:p.Ile994Val	1	0	0	0	VUS	1
ABCA4	NM_000350.2:c.3376C>G	NP_000341.2:p.Leu1126Val	1	0	0	0	VUS	1
ABCA4	NM_000350.2:c.6041T>C	NP_000341.2:p.Met2014Thr	1	0	0	0	VUS	1
GUCA1A	NM_000409.3:c.572_586dup	NP_000400.2:p.Glu191_Glu195dup	0	1	0	0	VUS	1
SAG	NM_000541.4:c.31G>A	NP_000532.2:p.Glu11Lys	0	1	0	0	VUS	1
IMPDH1	NM_000883.3:c.443C>T	NP_000874.2:p.Thr148Met	0	0	0	1	VUS	1
CERKL	NM_001030311.2:c.1358G>A	NP_001025482.1:p.Gly453Glu	0	0	0	1	VUS	1
RPGR	NM_001034853.1:c.1481G>T	NP_001030025.1:p.Gly494Val	0	0	0	1	VUS	1
FSCN2	NM_001077182.2:c.1085C>T	NP_001070650.1:p.Ala362Val	0	1	0	0	VUS	1
CNGB1	NM_001286130.1:c.1330G>C	NP_001273059.1:p.Glu444Gln	0	1	0	0	VUS	1
CNGB1	NM_001286130.1:c.3385C>T	NP_001273059.1:p.Arg1129Trp	0	1	0	0	VUS	1
COL9A1	NM_001851.4:c.904G>A	NP_001842.3:p.Gly302Ser	0	0	0	1	VUS	1
PRPF3	NM_004698.2:c.1481C>T	NP_004689.1:p.Thr494Met	0	0	0	1	VUS	1
WFS1	NM_006005.2:c.1597C>T	NP_005996.2:p.Pro533Ser	0	0	0	1	VUS	1
WFS1	NM_006005.2:c.472G>A	NP_005996.2:p.Glu158Lys	0	0	0	1	VUS	1

RP1	NM_006269.1:c.2497T>C	NP_006260.1:p.Phe833Leu	0	1	0	0	VUS	1
RP1	NM_006269.1:c.615+3G>A	-	0	1	0	0	VUS	1
PNPLA6	NM_006702.4:c.3058C>T	NP_006693.3:p.Arg1020Cys	0	0	0	1	VUS	1
PRPF6	NM_012469.3:c.2812A>G	NP_036601.2:p.Lys938Glu	0	1	0	0	VUS	1
PRPF31	NM_015629.3:c.902T>C	NP_056444.3:p.Leu301Pro	0	0	0	1	VUS	1
CNGB3	NM_019098.4:c.1534A>G	NP_061971.3:p.Ile512Val	0	1	0	0	VUS	1
NYX	NM_022567.2:c.505A>G	NP_072089.1:p.Asn169Asp	0	0	0	1	VUS	1
BBS1	NM_024649.4:c.1205T>C	NP_078925.3:p.Leu402Pro	0	0	0	1	VUS	1
CEP290	NM_025114.3:c.43C>A	NP_079390.3:p.Pro15Thr	0	0	0	1	VUS	1
CDHR1	NM_033100.3:c.562G>A	NP_149091.1:p.Gly188Ser	0	0	0	1	VUS	1
OPA1	NM_130837.2:c.1976A>T	NP_570850.2:p.Glu659Val	0	0	0	1	VUS	1
KCNV2	NM_133497.3:c.1148G>T	NP_598004.1:p.Arg383Leu	0	0	0	1	VUS	1
RDH12	NM_152443.2:c.806C>G	NP_689656.2:p.Ala269Gly	0	0	0	1	VUS	1
RP1L1	NM_178857.5:c.2561C>T	NP_849188.4:p.Pro854Leu	0	0	0	1	VUS	1
USH2A	NM_206933.2:c.12332C>T	NP_996816.2:p.Ser4111Phe	0	1	0	0	VUS	1
USH2A	NM_206933.2:c.5051C>T	NP_996816.2:p.Pro1684Leu	0	0	0	1	VUS	1
USH2A	NM_206933.2:c.5612G>A	NP_996816.2:p.Gly1871Asp	0	0	0	1	VUS	1
USH2A	NM_206933.2:c.7067A>G	NP_996816.2:p.Asn2356Ser	0	0	0	1	VUS	1
USH2A	NM_206933.2:c.841A>C	NP_996816.2:p.Thr281Pro	0	1	0	0	VUS	1
HMCN1	NM_031935.2:c.14609-1G>A	-	0	0	0	0	VUS	0
PRPF8	ENST00000572621.1:c.5881A>G	ENSP00000460348.1:p.Ile1961Val	0	0	0	0	VUS	0
GUCY2D	NM_000180.3:c.187G>A	NP_000171.1:p.Ala63Thr	0	0	0	0	VUS	0
GUCY2D	NM_000180.3:c.2407G>C	NP_000171.1:p.Asp803His	0	0	0	0	VUS	0
PDE6B	NM_000283.3:c.272G>T	NP_000274.2:p.Arg91Leu	0	0	0	0	VUS	0
PRPH2	NM_000322.4:c.660_665del	NP_000313.2:p.Pro221_Cys222del	0	0	0	0	VUS	0
PRPH2	NM_000322.4:c.781C>T	NP_000313.2:p.Leu261Phe	0	0	0	0	VUS	0
RS1	NM_000330.3:c.121G>A	NP_000321.1:p.Asp41Asn	0	0	0	0	VUS	0
CA4	NM_000717.4:c.341T>C	NP_000708.1:p.Leu114Ser	0	0	0	0	VUS	0
IMPDH1	NM_000883.3:c.1475G>A	NP_000874.2:p.Arg492Gln	0	0	0	0	VUS	0
GDF6	NM_001001557.2:c.125G>T	NP_001001557.1:p.Gly42Val	0	0	0	0	VUS	0
C2orf71	NM_001029883.2:c.1094_1102del	NP_001025054.1:p.Leu365_Lys367de	0	0	0	0	VUS	0
KLHL7	NM_001031710.2:c.4G>A	NP_001026880.2:p.Ala2Thr	0	0	0	0	VUS	0
FSCN2	NM_001077182.2:c.1071_1073del	NP_001070650.1:p.Lys357del	0	0	0	0	VUS	0

FSCN2	NM_001077182.2:c.454G>A	NP_001070650.1:p.Val152Met	0	0	0	0	VUS	0
CNGA3	NM_001079878.1:c.1147T>C	NP_001073347.1:p.Ser383Pro	0	0	0	0	VUS	0
CNGA3	NM_001079878.1:c.757C>G	NP_001073347.1:p.Pro253Ala	0	0	0	0	VUS	0
AIPL1	NM_014336.5:c.140C>G	NP_055151.3:p.Thr47Arg	0	0	0	0	VUS	0
AIPL1	NM_014336.5:c.937G>T	NP_055151.3:p.Ala313Ser	0	0	0	0	VUS	0
EYS	NM_001142800.1:c.154G>C	NP_001136272.1:p.Asp52His	0	0	0	0	VUS	0
EYS	NM_001142800.1:c.25C>A	NP_001136272.1:p.Leu9Met	0	0	0	0	VUS	0
EYS	NM_001142800.1:c.3731C>T	NP_001136272.1:p.Thr1244Ile	0	0	0	0	VUS	0
PDZD7	NM_001195263.1:c.893A>G	NP_001182192.1:p.Lys298Arg	0	0	0	0	VUS	0
CACNA1F	NM_001256789.1:c.4009-3C>G	-	0	0	0	0	VUS	0
ADIPOR1	NM_001290557.1:c.1058A>G	NP_001277486.1:p.Tyr353Cys	0	0	0	0	VUS	0
CNGA3	NM_001298.2:c.1789G>A	NP_001289.1:p.Ala597Thr	0	0	0	0	VUS	0
CNGA3	NM_001298.2:c.79C>T	NP_001289.1:p.Arg27Cys	0	0	0	0	VUS	0
GUCA1A	NM_001319061.1:c.149C>T	NP_001305990.1:p.Pro50Leu	0	0	0	0	VUS	0
GUCA1B	NM_002098.5:c.357+3del	-	0	0	0	0	VUS	0
OFD1	NM_003611.2:c.87T>G	NP_003602.1:p.Asp29Glu	0	0	0	0	VUS	0
UNC119	NM_005148.3:c.62C>T	NP_005139.1:p.Ser21Leu	0	0	0	0	VUS	0
CACNA1F	NM_005183.2:c.4294A>T	NP_005174.2:p.Ile1432Phe	0	0	0	0	VUS	0
CACNA1F	NM_005183.2:c.5617C>T	NP_005174.2:p.Arg1873Cys	0	0	0	0	VUS	0
PROM1	NM_006017.2:c.557G>A	NP_006008.1:p.Arg186Gln	0	0	0	0	VUS	0
RP1	NM_006269.1:c.60A>C	NP_006260.1:p.Gln20His	0	0	0	0	VUS	0
MERTK	NM_006343.2:c.1274T>A	NP_006334.2:p.Val425Glu	0	0	0	0	VUS	0
MERTK	NM_006343.2:c.2209G>A	NP_006334.2:p.Val737Ile	0	0	0	0	VUS	0
MERTK	NM_006343.2:c.2435A>G	NP_006334.2:p.Tyr812Cys	0	0	0	0	VUS	0
PRPF8	NM_006445.3:c.6473A>C	NP_006436.3:p.His2158Pro	0	0	0	0	VUS	0
PRPF31	NM_015629.3:c.855+5G>A	-	0	0	0	0	VUS	0
IMPG2	NM_016247.3:c.1300C>T	NP_057331.2:p.Pro434Ser	0	0	0	0	VUS	0
IMPG2	NM_016247.3:c.1460A>T	NP_057331.2:p.His487Leu	0	0	0	0	VUS	0
IMPG2	NM_016247.3:c.1582A>G	NP_057331.2:p.Ile528Val	0	0	0	0	VUS	0
IMPG2	NM_016247.3:c.2731_2733del	NP_057331.2:p.Met911del	0	0	0	0	VUS	0
SPATA7	NM_018418.4:c.770G>A	NP_060888.2:p.Arg257His	0	0	0	0	VUS	0
CDH23	NM_022124.5:c.4231G>A	NP_071407.4:p.Glu1411Lys	0	0	0	0	VUS	0
NYX	NM_022567.2:c.1003C>T	NP_072089.1:p.Arg335Cys	0	0	0	0	VUS	0

PANK2	NM_024960.5:c.338A>T	NP_079236.3:p.Asn113Ile	0	0	0	0	VUS	0
CEP290	NM_025114.3:c.4081C>G	NP_079390.3:p.Leu1361Val	0	0	0	0	VUS	0
CEP290	NM_025114.3:c.6970C>T	NP_079390.3:p.Leu2324Phe	0	0	0	0	VUS	0
RAX2	NM_032753.3:c.466G>A	NP_116142.1:p.Ala156Thr	0	0	0	0	VUS	0
CDHR1	NM_033100.3:c.1554-3C>A	-	0	0	0	0	VUS	0
CDHR1	NM_033100.3:c.1589C>G	NP_149091.1:p.Thr530Ser	0	0	0	0	VUS	0
COL11A1	NM_080629.2:c.1718A>G	NP_542196.2:p.Gln573Arg	0	0	0	0	VUS	0
COL11A1	NM_080629.2:c.3961G>A	NP_542196.2:p.Gly1321Ser	0	0	0	0	VUS	0
COL11A1	NM_080629.2:c.4838C>A	NP_542196.2:p.Thr1613Asn	0	0	0	0	VUS	0
HGSNAT	NM_152419.2:c.1237C>T	NP_689632.2:p.Pro413Ser	0	0	0	0	VUS	0
HGSNAT	NM_152419.2:c.726C>T	NP_689632.2:p.Ser242%3D	0	0	0	0	VUS	0
RP1L1	NM_178857.5:c.292G>A	NP_849188.4:p.Asp98Asn	0	0	0	0	VUS	0
RP1L1	NM_178857.5:c.329C>G	NP_849188.4:p.Pro110Arg	0	0	0	0	VUS	0
RP1L1	NM_178857.5:c.4630C>T	NP_849188.4:p.Arg1544Cys	0	0	0	0	VUS	0
CRB1	NM_201253.2:c.430T>G	NP_957705.1:p.Phe144Val	0	0	0	0	VUS	0
CRB1	NM_201253.2:c.498_506del	NP_957705.1:p.Ile167_Gly169del	0	0	0	0	VUS	0
CRB1	NM_201253.2:c.614T>C	NP_957705.1:p.Ile205Thr	0	0	0	0	VUS	0
USH2A	NM_206933.2:c.10364G>T	NP_996816.2:p.Ser3455Ile	0	0	0	0	VUS	0
USH2A	NM_206933.2:c.13099G>A	NP_996816.2:p.Val4367Ile	0	0	0	0	VUS	0
USH2A	NM_206933.2:c.15586A>C	NP_996816.2:p.Thr5196Pro	0	0	0	0	VUS	0
USH2A	NM_206933.2:c.5266G>A	NP_996816.2:p.Val1756Ile	0	0	0	0	VUS	0

Supplementary Table 16. The 13 VUS variants with new evidences found after reassessment.

Gene	HGVSc	HGVSp	Alleles	Gene_inheritance_pattern	Inheritance_data
ABCA4	NM_000350.2:c.2980A>G	NP_000341.2:p.Ile994Val	R_33	recessive	no AF (sporadic/AR)
ABCA4	NM_000350.2:c.5383T>G	NP_000341.2:p.Leu1795Val	R_33	recessive	no AF (sporadic/AR)
PRPF3	NM_004698.2:c.1481C>T	NP_004689.1:p.Thr494Met	D_3	dominant	AD
USH2A	NM_206933.2:c.7067A>G	NP_996816.2:p.Asn2356Ser	R_30	recessive	sporadic
HK1	NM_033497.2:c.1346C>T	NP_277032.1:p.Ser449Leu	D_3	dominant	no AF (sporadic/AR)
GUCY2D	NM_000180.3:c.2705T>C	NP_000171.1:p.Val902Ala	D_3	dominant	AD
BBS1	NM_024649.4:c.1205T>C	NP_078925.3:p.Leu402Pro	R_33	recessive	AR
RPGR	NM_001034853.1:c.379A>G	NP_001030025.1:p.Arg127Gly	X_3	X-linked	no AF (sporadic/AR)
BEST1	NM_001139443.1:c.671A>G	NP_001132915.1:p.Tyr224Cys	D_3	dominant	AD
IMPG2	NM_016247.3:c.2872A>G	NP_057331.2:p.Ser958Gly	D_3	dominant	AD
USH2A	NM_206933.2:c.12332C>T	NP_996816.2:p.Ser4111Phe	R_33	recessive	no AF (sporadic/AR)
USH2A	NM_206933.2:c.841A>C	NP_996816.2:p.Thr281Pro	R_33	recessive	no AF (sporadic/AR)
BEST1	NM_004183.3:c.828C>G	NP_004174.1:p.Phe276Leu	gh	dominant	AD

Segregation study	Final Status	Conclusion
Unavailable	Evidence supporting pathogenicity	To further be considered
Unavailable	Evidence supporting pathogenicity	To further be considered
Unavailable	Evidence supporting pathogenicity	To further be considered
Unavailable	Evidence supporting pathogenicity	Reclassified
De novo variant	Evidence supporting pathogenicity	Reclassified
Unavailable	Evidence supporting pathogenicity	To further be considered
Variant cosegregates with disease in two affected sisters	Evidence supporting pathogenicity	Reclassified
Unavailable	Evidence supporting pathogenicity	To further be considered
Unavailable	Evidence supporting pathogenicity	Reclassified
Affected father with variant	Evidence supporting pathogenicity	Reclassified
Yes, variants in trans	Evidence supporting pathogenicity	To further be considered
Yes, variants in trans	Evidence supporting pathogenicity	To further be considered
Healthy uncle does not have it	Evidence supporting pathogenicity	To further be considered

Evidences	ACMG Classification
Consistent with phenotype and reported as likely pathogenic in VarSome	VUS
Consistent with phenotype and reported as likely pathogenic in VarSome	VUS
Consistent with phenotype, and inheritance pattern and reported pathogenic in VarSome	VUS
Same amino acid change previously reported pathogenic	Likely pathogenic
De novo variant	Likely pathogenic
Consistent with phenotype and inheritance pattern and reported as likely pathogenic in VarSome	VUS
Variants cosegregates with disease in two affected sisters.	Likely pathogenic
Consistent with phenotype and reported as likely pathogenic in Varsome and ClinVar	VUS
Same amino acid change previously reported pathogenic	Likely pathogenic
Cosegregates with the disease in the affected father	Likely pathogenic
Consistent with phenotype and in trans with VUS variant (NM_206933.2:c.841A>C)	VUS
Consistent with phenotype and in trans with VUS variant (NM_206933.2:c.12332C>T)	VUS
Consistent with phenotype and inheritance pattern	VUS

ACMG Criteria
(ACMG: PM2 Moderate; PP3 Supporting)
(ACMG: PM2 Moderate; PP3 Supporting)
(ACMG: PM2 Moderate, PP3 Supporting; PP5 Supporting)
(ACMG: PS1 Strong; PM2 Moderate; PP3 Supporting)
(ACMG: PM2 Moderate; PM6 Moderate; PP3 Supporting; PP5 Supporting)
(ACMG: PM2 Moderate; PM5 Moderate; PP5 Supporting)
(ACMG: PM2: Moderate; PP1 Supporting; PP2 Supporting; PP3 Supporting)
(ACMG: PM2 Moderate; PP3 Supporting)
(ACMG: PS2 Strong; PM2 Moderate; PP3 Supporting)
(ACMG: PM2 Moderate; PP1 Supporting; PP2 Supporting; PP3 Supporting)
(ACMG: PM2 Moderate, PP2 Supporting, BP4 Supporting)
(ACMG: PM2 Moderate, PP2 Supporting, PP3 Supporting)
(ACMG: PM2 Moderate; PP3 Supporting)

Supplementary Table 17. List of non-IRD sub cohort of diseases and diagnosis distribution

	Diagnostic			
**Diseases**	**Non solved**	**Partially solved**	**Solved**	**VUS**
Corneal dystrophy	15	0	16	9
Encephalopathies ID Epilepsy	91	5	27	48
Eye malformation	39	7	27	16
Hearing loss	16	4	14	24
Metabolic	101	4	34	15
Mixed conditions	117	3	35	16
Myopathies	53	3	16	21
Nephropathies	27	3	13	6
Neurodegeneration	57	2	12	14
Optic neuropathy	50	1	21	7
Peripheral neuropathies	38	1	22	25
Polymalformative syndromes	133	3	33	25
**TOTAL**	**737**	**36**	**270**	**226**

Supplementary Table 18. Virtual panels used and size in number of genes

Virtual panels	Panel size (genes)
Vitreoretinopathy	5
Stickler	8
Usher	10
Optical Atrophy	15
Bardet-Biedl	20
Ocular Albinism	38
Retinal Dystrophy / Macular Dystrophy	54
Syndromic Retinal Dystrophy	106
Non-Syndromic Retinal Dystrophy	136
Retinal Dystrophy	205
RetNet	229
Customized-FJD	237

Supplementary Figure 1. Proportion of bases at coverage in IRD for TSO and CES.

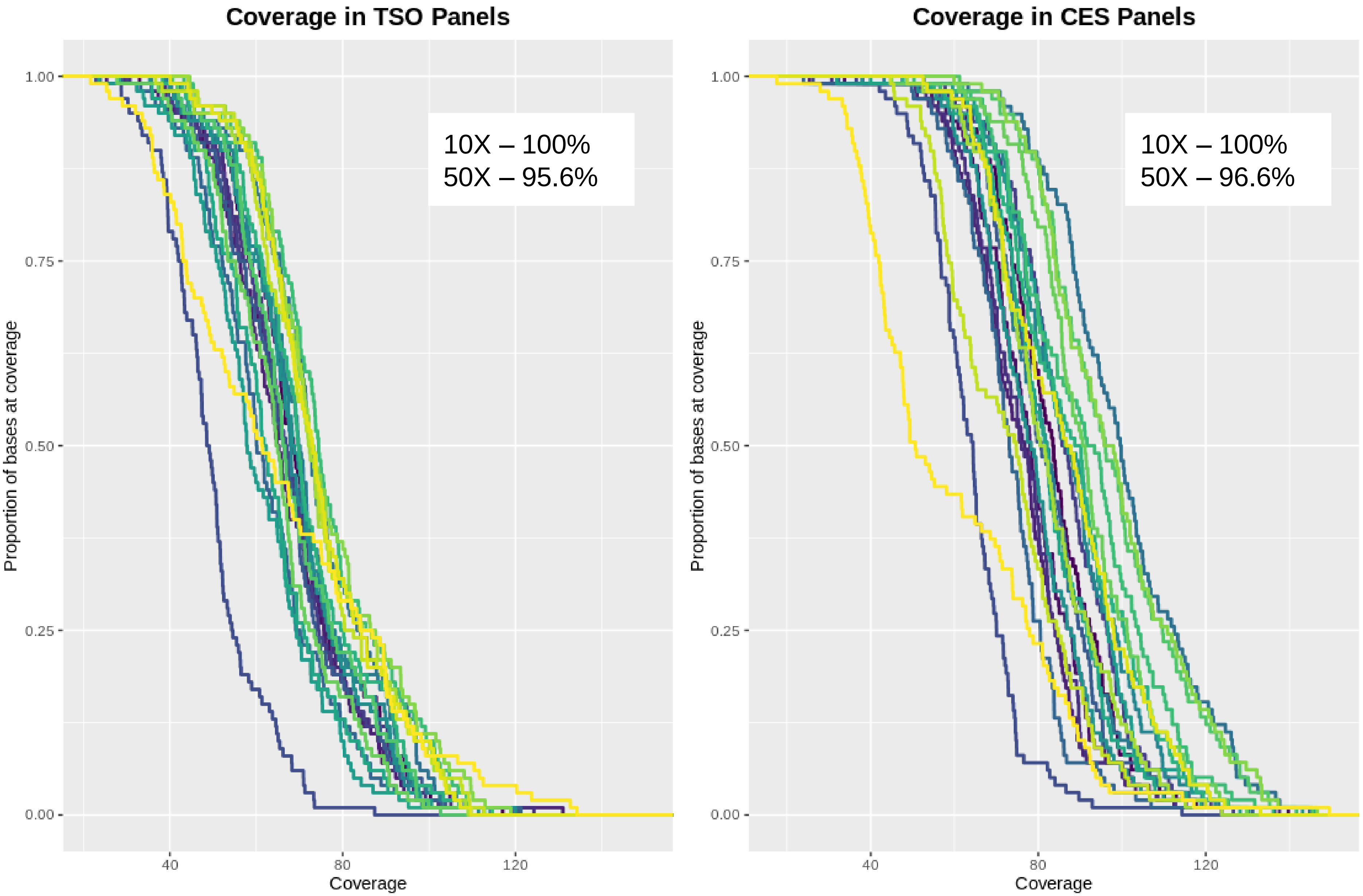

Supplementary Figure 2. Number of variants detected for TSO and CES panels.

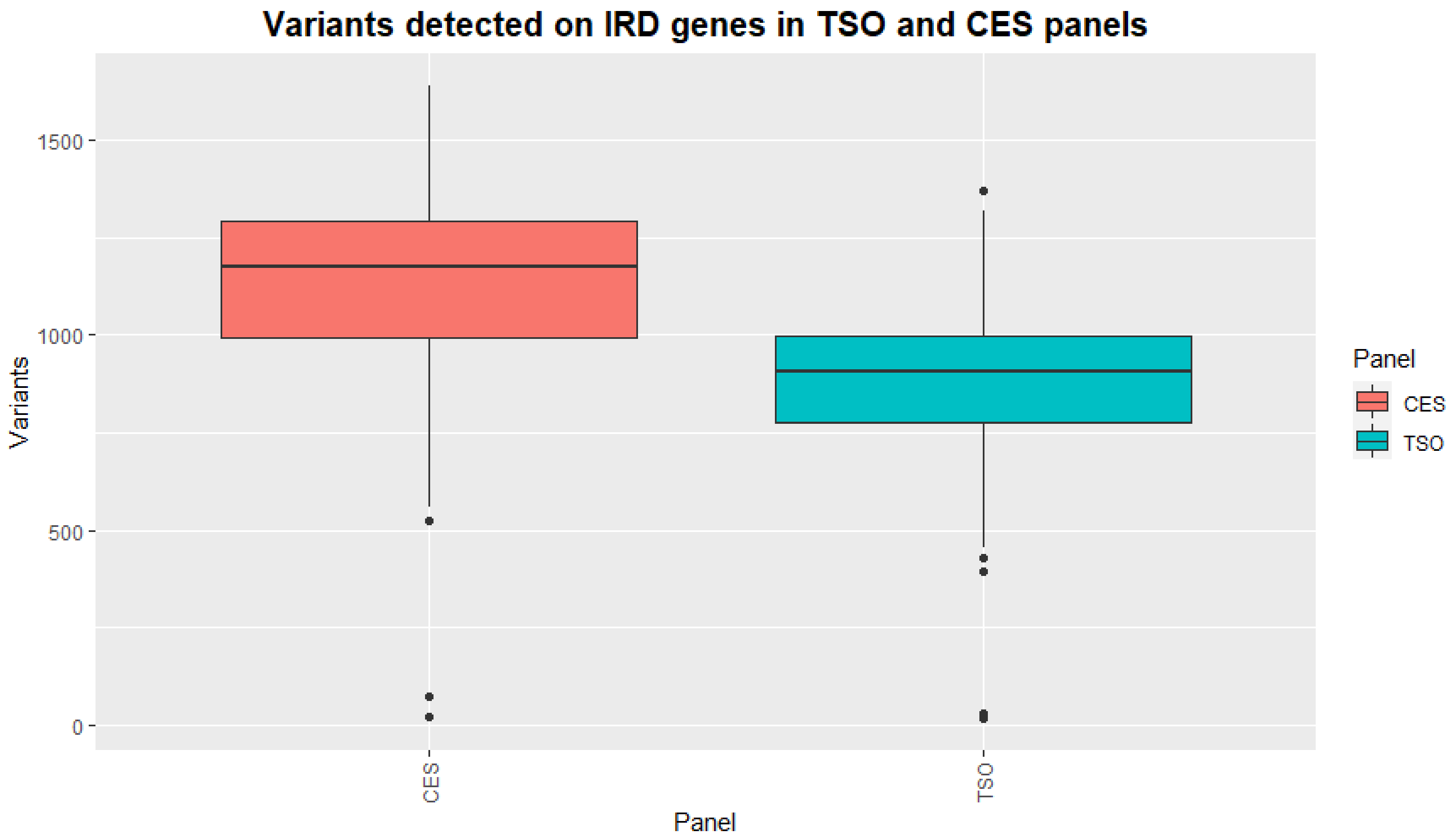

Supplementary Figure 3. ExACpLI score distribution for all VUS and Pathogenic variants (A) and for Loss of Function (LoF) VUS and pathogenic variants (B).

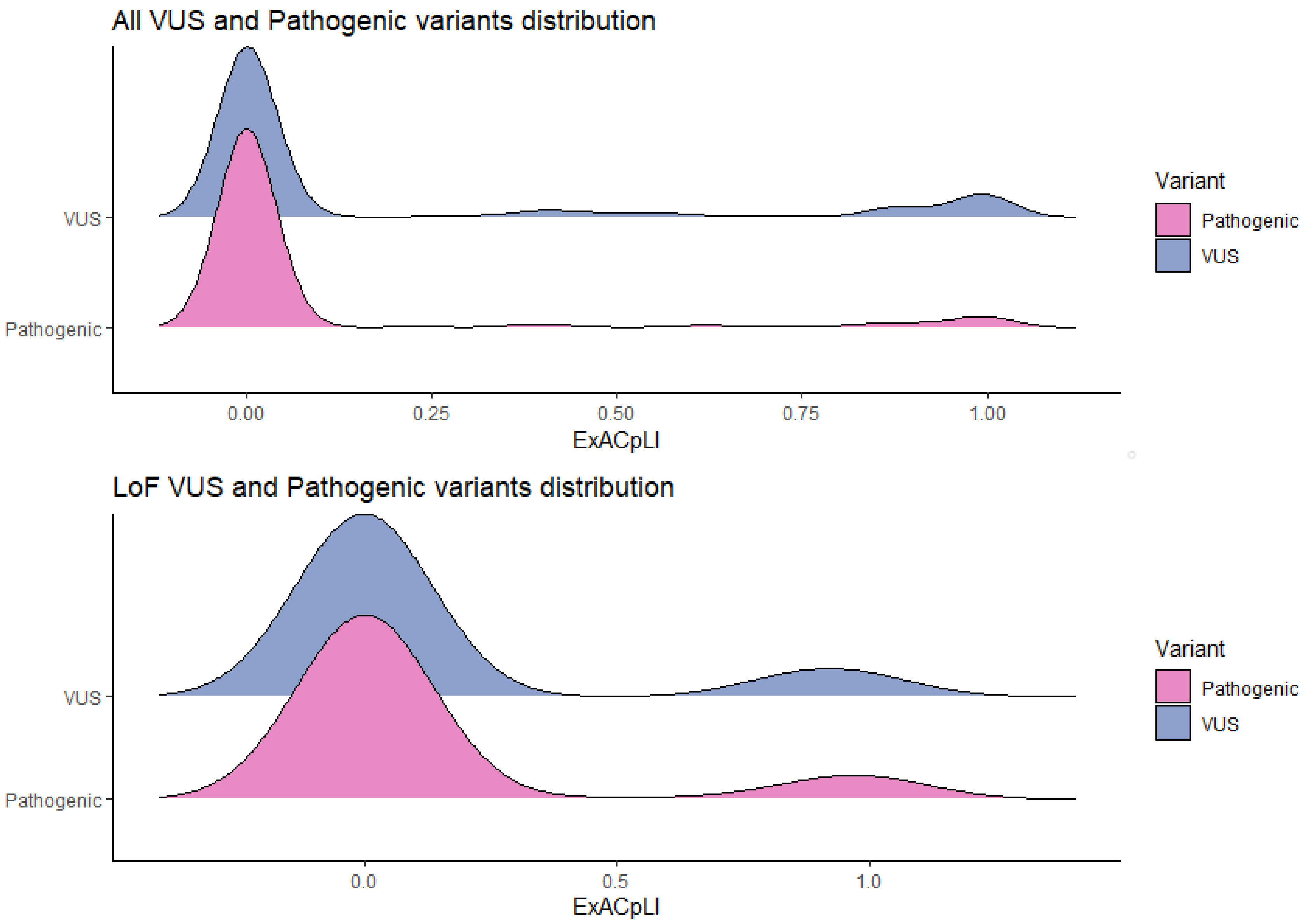

Supplementary Figure 4. CCR scores across VUS and pathogenic variants.

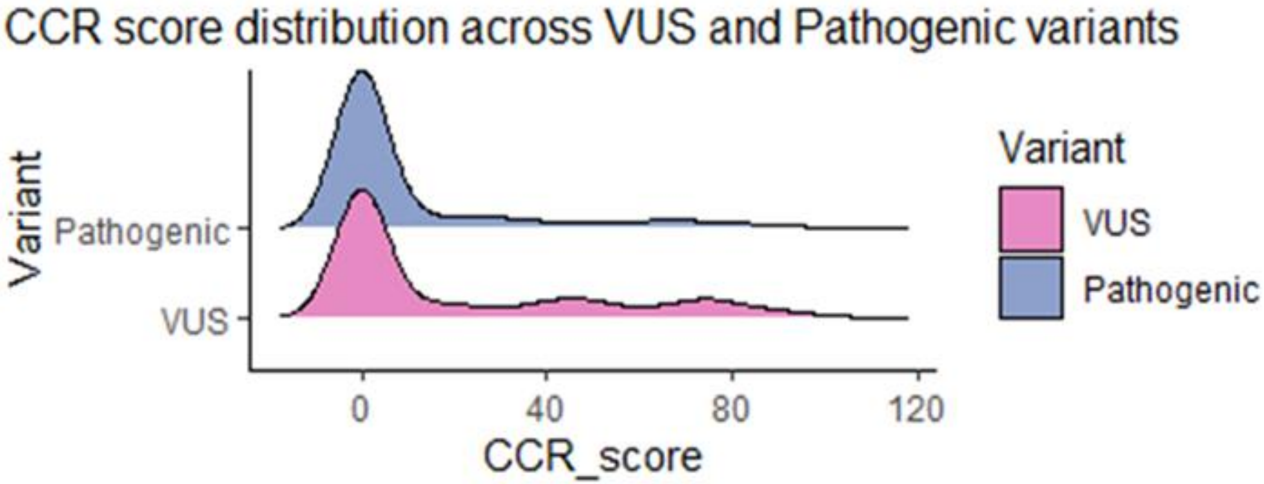

Supplementary Figure 5. Genes overlapping between TSO-CES clinical exome panels and IRD genes.

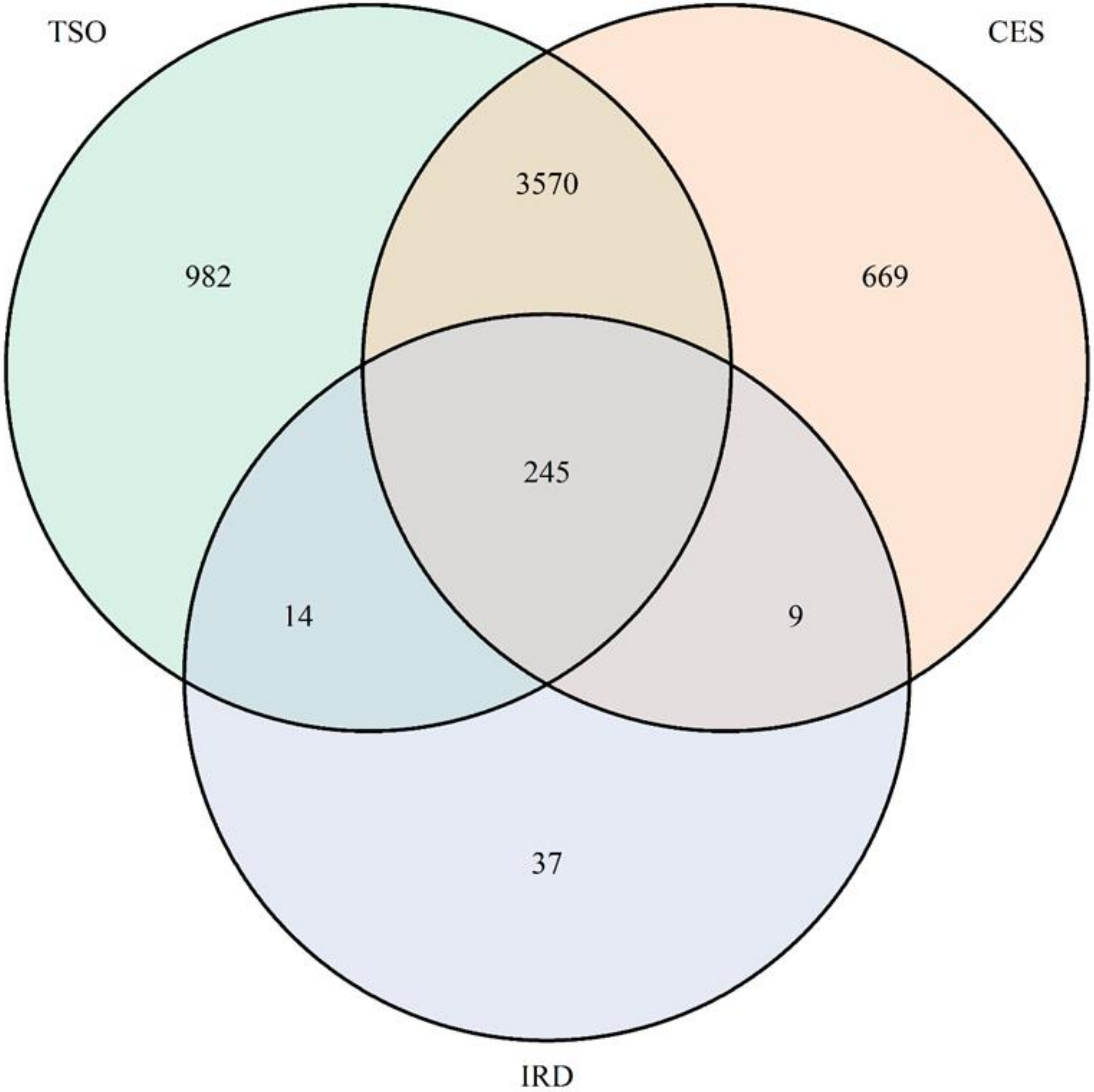

Supplement: Supplementary file 1 — Supplementary Information [file 41525_2021_182_MOESM1_ESM.pdf]
